# Impact of a mindfulness‐based intervention on neurobehavioral functioning and its association with large‐scale brain networks in preterm young adolescents

**DOI:** 10.1111/pcn.13675

**Published:** 2024-05-17

**Authors:** Vanessa Siffredi, Maria Chiara Liverani, Natalia Fernandez, Lorena G. A. Freitas, Cristina Borradori Tolsa, Dimitri Van De Ville, Petra Susan Hüppi, Russia Ha‐Vinh Leuchter

**Affiliations:** ^1^ Division of Development and Growth, Department of Paediatrics, Gynaecology and Obstetrics Geneva University Hospitals and University of Geneva Geneva Switzerland; ^2^ Neuro‐X Institute École polytechnique fédérale de Lausanne Geneva Switzerland; ^3^ Department of Radiology and Medical Informatics, Faculty of Medicine University of Geneva Geneva Switzerland; ^4^ SensoriMotor, Affective and Social Development Laboratory, Faculty of Psychology and Educational Sciences University of Geneva Geneva Switzerland

**Keywords:** adolescence, dynamic functional connectivity, intervention, mindfulness, preterm

## Abstract

**Aim:**

Adolescents born very preterm (VPT; <32 weeks of gestation) face an elevated risk of executive, behavioral, and socioemotional difficulties. Evidence suggests beneficial effects of mindfulness‐based intervention (MBI) on these abilities. This study seeks to investigate the association between the effects of MBI on executive, behavioral, and socioemotional functioning and reliable changes in large‐scale brain networks dynamics during rest in VPT young adolescents who completed an 8‐week MBI program.

**Methods:**

Neurobehavioral assessments and resting‐state functional magnetic resonance imaging were performed before and after MBI in 32 VPT young adolescents. Neurobehavioral abilities in VPT participants were compared with full‐term controls. In the VPT group, dynamic functional connectivity was extracted by using the innovation‐driven coactivation patterns framework. The reliable change index was used to quantify change after MBI. A multivariate data‐driven approach was used to explore associations between MBI‐related changes on neurobehavioral measures and temporal brain dynamics.

**Results:**

Compared with term‐born controls, VPT adolescents showed reduced executive and socioemotional functioning before MBI. After MBI, a significant improvement was observed for all measures that were previously reduced in the VPT group. The increase in executive functioning, only, was associated with reliable changes in the duration of activation of large‐scale brain networks, including frontolimbic, amygdala‐hippocampus, dorsolateral prefrontal, and visual networks.

**Conclusion:**

The improvement in executive functioning after an MBI was associated with reliable changes in large‐scale brain network dynamics during rest. These changes encompassed frontolimbic, amygdala‐hippocampus, dorsolateral prefrontal, and visual networks that are related to different executive processes including self‐regulation, attentional control, and attentional awareness of relevant sensory stimuli.

Very preterm (VPT) birth, as defined by a birth occurring before 32 weeks of gestation, affects the developmental trajectory of the brain. It is consequently associated with an increased risk of cognitive and behavioral difficulties across the lifespan.^1^ In a review study, Johnson and Marlow^2^ found that when diagnostic psychiatric evaluations were employed, studies reported a threefold to fourfold increased risk for developing disorders during middle childhood. The most commonly observed were the inattentive subtype of attention‐deficit/hyperactivity disorder, anxiety disorders, and autism spectrum disorder.^2^ In light of these findings, they introduced the concept of the ‘preterm behavioral phenotype’, which is characterized by an augmented susceptibility to disorders and subclinical symptoms related to attention and executive functions, anxiety, and socioemotional difficulties.

Resting‐state functional connectivity (FC) is now a commonly used technique for mapping large‐scale networks in the human brain. It is characterized by the temporal dependence of neuronal activity patterns of anatomically separated brain regions occurring in the absence of goal‐directed activity and stimulation.^3^ A set of functionally connected regions with synchronous and spontaneous neuronal activity observed in the resting state is referred to as resting‐state networks.^4^ In VPT individuals from birth to adulthood, atypical FC has been observed using both static and dynamic FC.^5^ Importantly, these alterations have been associated with the symptoms described as the preterm behavioral phenotype, including executive, behavioral, and socioemotional difficulties in VPT children, adolescents, and adults.^6^, ^7^, ^8^

More specifically, static FC studies using a seed‐based correlation approach have shown altered FC of various brain regions in infants, children, adolescents, and adults born prematurely including the superior temporal, amygdala, posteromedial and lateral parietal, prefrontal, and lateral and superior sensorimotor regions.^7^ In VPT infants, overall cognitive scores at 6 months were predicted by medial prefrontal cortex‐executive control connectivity strength.^9^ In VPT children and adults, increased and decreased FC of the amygdala with other brain regions including posterior cingulate cortex, precuneus, and superior temporal areas and thalamus have been associated with the capacity to recognize facial expression of anger and with social problems.^7^, ^10^, ^11^ Similarly, static FC studies using whole‐brain data‐driven approaches have found atypical resting‐state networks associated with prematurity across the lifespan, especially FC of the salience, default mode, and frontal networks.^6^, ^12^ In VPT children, Mossad *et al*.^13^ showed that increased connectivity in the default mode network at the age of 6 was linked to social and working memory difficulties by the age of 8. Moreover, in VPT children and adolescents, intelligence quotient or poorer executive function abilities have been partially linked to FC strength between sensorimotor areas and those involved in higher‐order cognitive functions (i.e. salience, central executive, and dorsal attention networks).^14^

Studies using dynamic FC—allowing identification of meaningful variations over time in FC between different brain regions—are still sparse in the VPT population and, to our knowledge, only conducted in VPT infants and children so far. Nevertheless, the findings are consistent with static FC with different patterns of brain dynamics between VPT groups and full‐term (FT) controls.^15^, ^16^ In FT controls, reduced involvement in specific brain networks correlated with improved socioemotional abilities; while in the VPT group, enhanced socioemotional abilities were associated with the coordination of activity across various functional networks, i.e. coupling duration between different pairs of networks.^8^

Given the significant long‐term impact of deficits reflecting the preterm phenotype, greater efforts have focused on developing potential ways to reduce these difficulties. Mindfulness, commonly defined as the ongoing monitoring of present‐moment experience while attending to it in an open and accepting way and without judgment,^17^ is of particular interest. Mindfulness‐based intervention (MBI), designed to improve mindfulness skills through diverse practices, has been linked to enhanced executive, behavioral and socioemotional functioning in typically developing children and adolescents,^18^, ^19^ as well as in clinical pediatric populations.^20^, ^21^ Consistent with enhancements in behavioral outcomes, research indicates that MBI may induce FC changes. A recent review of the literature showed the wide impact of mindfulness and MBI on brain FC in healthy and clinical populations, especially FC related to prefrontal, rostral anterior cingulate, amygdala, and visual regions.^22^ Comparable results have been found during adolescence in typically developing as well as in clinical populations.^23^, ^24^ There is limited research on the impact of mindfulness on dynamic FC. Nevertheless, a recent study found increased FC between the default mode network and the salience network in adults after 1 month of mindfulness meditation compared with a control intervention.^25^ Moreover, in children, adolescents, and adults, consistent findings showed that individuals with higher disposition to mindfulness or a mindfulness trait (i.e. corresponding to the average frequency with which individuals experience states of mindfulness) transitioned between brain states more frequently than those with low mindfulness disposition.^26^, ^27^

In VPT young adolescents, a recent randomized controlled trial demonstrated advantages from an 8‐week MBI on executive, behavioral, and socioemotional functioning.^28^ More precisely, the results suggest a beneficial effect of MBI on executive, behavioral, and socioemotional functioning in VPT young adolescents, as assessed through parent questionnaires. In addition, increased executive abilities were observed using a computerized task, reflecting improved processing speed following MBI. Building on our prior investigation and seeking a deeper comprehension of the neural underpinnings of neurobehavioral changes, the objective of this study is to evaluate the association between the benefits of MBI on neurobehavioral functioning and potential changes in large‐scale brain dynamics, employing dynamic FC. To achieve this, we initially compared VPT and FT young adolescents across various measures of executive, behavioral, and socioemotional measures. Next, we assessed the benefit of the MBI using a before/after design on neurobehavioral measures that showed significant differences between the VPT and the FT groups (i.e. significant reduction in neurobehavioral symptoms in the VPT group compared with their FT peers). Of note, only VPT young adolescents (and not FT controls) completed the MBI. Finally, we explored associations between reliable change after MBI on neurobehavioral measures and reliable change after MBI on temporal characteristics of large‐scale brain networks.

Recent studies indicate that static FC may oversimplify the characterization of resting‐state activity by ignoring its inherent dynamic fluctuations.^29^, ^30^, ^31^ To address this limitation, dynamic approaches have been developed, offering the potential to identify significant temporal variations in FC among distinct brain regions. Moreover, a dynamic FC approach holds promise to provide information for both the identification of markers of cognitive development in the VPT population,^8^, ^15^, ^16^ as well for the identification of mindfulness‐related changes in brain functions.^26^ Among the multiple existing methods to investigate dynamic FC,^29^ the innovation‐driven coactivation patterns (iCAPs) framework allows capture of transient activations through spatiotemporal functional magnetic resonance imaging (fMRI) deconvolution, and then defines iCAPs network containing all brain voxels with the same transient behavior.^32^, ^33^ This approach aligns conceptually with the hypothesis of discrete shifts between brain meta‐states,^34^ although states in the iCAPs framework can be temporally overlapping. In contrast to other dynamic FC approaches (e.g. sliding windows), iCAPs can detect instantaneous transients of activity of areas clustered together based on their activation pattern. This enables the dissection of brain activity patterns that temporally coincide by identifying consistent spatial patterns at the onset of deactivation or activation.^33^

## Methods

### Participants

This study uses data from the Mindful Preterm Teens Study, a randomized controlled trial.^35^ A total of 63 VPT young adolescents (born before 32 gestational weeks) were enrolled in the study. Fifty‐two of them completed the MBI and the neurobehavioral assessment before and after the intervention. The participants, ranging from 10 to 14 years, were born between January 2003 and December 2008 at the Geneva University Hospital, Switzerland. They were under the care of the Division of Child Development and Growth at the Geneva University Hospital. Exclusions for VPT young adolescents involved an intelligence quotient below 70, sensory or physical disabilities (such as cerebral palsy, blindness, or hearing loss), or an inadequate proficiency in French. Of the 52 young adolescents, 39 completed an MRI scan both before and after the MBI. Seven were removed from the final analysis because of head motion, and a final sample of 32 VPT participants was finally included in the current resting‐state fMRI (rs‐fMRI) analyses (Supplementary Fig. S1).

Moreover, 24 FT and typically developing young adolescents aged between 10 and 14 years were recruited through the community and completed the neurobehavioral assessment and MRI scan (similar to the assessment completed by the VPT group before the MBI).

The current study conforms to the provisions of the Declaration of Helsinki.

### Mindfulness‐based intervention

The MBI was specifically created for the Mindful Preterm Teens Study and consisted of 8 weekly sessions in groups of up to 8 participants, lasting 90 min (for a detailed description of the MBI, see Supplementary Methods and Supplementary Table S1)^35^.

### Neurobehavioral outcome measures

Participants’ executive, behavioral, and socioemotional functioning were assessed. Table 1 provides an overview of the assessment (see Supplementary Table S2 for a comprehensive description of each measure). Importantly, all outcome measures assessed and utilized in our prior randomized controlled trial were also retained in the current study. The rationale for choosing these outcome measures is described thoroughly in the feasibility study and the effectiveness of the randomized controlled trial.^28^, ^35^

**Table 1:** Neurobehavioral outcome measures and scores

Domains	Modalities	Measures	Scores
Executive functions			
	Parent‐reported questionnaires		
		Behavior Rating Inventory of Executive Function, parent version (BRIEF^36^) Evaluates executive functions in everyday life	
		Global Executive Composite (GEC), Behavioral Regulation Index (BRI), Metacognition index (MI). Standardized T scores were used (*M* = 50; SD = 10). Higher scores reflect increased difficulties in executive functioning	BRIEF GEC BRIEF BRI BRIEF MI
	Neuropsychological tests		
		Letter‐Number Sequencing (WISC‐V^37^)	
		Evaluates working memory. Standardized scores were used (*M* = 10; SD = 3). Higher standardized scores reflect higher working memory skills	Letter‐number sequencing
		Tempo Test Rekenen^38^	
		Evaluates arithmetic. The total raw score was used. Higher total scores reflect higher arithmetic skills	Tempo test
	Neurocognitive computerized tasks		
		Flanker Visual Filtering Task^39^	
		Evaluates attentional control and information processing speed Higher mean reaction times in the congruent condition reflect slower processing speed. Higher inhibition scores reflect increased difficulties in attentional control	Flanker processing speed Flanker inhibition
		Reality Filtering Task^40^	
		Evaluates reality filtering. The Temporal Context Confusion index was used as a reality filtering score. Higher TCC score indicate increased reality filtering difficulties	Reality filtering TCC
Behavior and socioemotional abilities			
	Parent‐reported questionnaires		
		Strengths and Difficulties Questionnaire, parent version (SDQ^41^)	
		Evaluates behavior and socioemotional abilities in everyday life. The SDQ total difficulty score was used. Higher SDQ total scores reflect increased behavioral and socioemotional difficulties	SDQ total
	Self‐reported questionnaires		
		KIDSCREEN‐27^42^	
		Evaluates health‐related quality of life. The total raw score was used. Higher total scores reflect increased quality of life and well‐being	KIDSCREEN total
		Social Goal Scale (SGS^43^)	
		Evaluates social responsiveness. The total raw score was used. Higher total scores reflect increased social goal in daily life	Social goal
		Self‐Compassion Scale – Short form (SCS^44^)	
		Evaluates self‐compassion. The total raw score was used. Higher total scores reflect increased self‐compassion	Self‐compassion
	Neuropsychological tests		
		Affect recognition (NEPSY‐II^45^)	
		Evaluates the ability to recognize facial emotional expressions. Standardized scores were used (*M* = 10; SD = 3). Higher scores reflect better affect recognition skills	Affect recognition
		Theory of Mind (NEPSY‐II^45^)	
		Evaluates the ability of mental functions and other people's perspectives. The total raw score was used. Higher scores reflect better theory of mind skills	Theory of mind

### MRI acquisition and preprocessing

#### MRI acquisition

MRI data were acquired using a Siemens 3T Magnetom Prisma scanner (Siemens, Erlangen, Germany) including structural T1‐weighted MP‐RAGE sequences, resting‐state functional images T2*‐weighted with a multislice gradient echo‐planar imaging sequence, and a fieldmap (see Supplementary Methods for a detailed description). During the rs‐fMRI sequence, young adolescents from the VPT and FT groups received identical instructions. They were asked to keep their eyes closed, try not to think about anything specific, and engage in mind wandering.

#### rs‐fMRI data preprocessing

rs‐fMRI data were preprocessed using SPM12 (Wellcome Department of Imaging Neuroscience) in MATLAB R2016a (The MathWorks, Inc.), following the preprocessing pipeline previously described (see Supplementary Methods for a detailed description).^36^, ^37^ In short, rs‐fMRI findings were spatially realigned and unwarped using the fieldmap images acquired, respectively. Functional images were then coregistered to their corresponding structural images in subject space. Structural images were segmented with the SPM12 segmentation algorithm, and a study‐specific template was generated using DARTEL^38^ that was used in the iCAPs framework described below. Finally, the first five rs‐fMRI scans were excluded. For each participant, preprocessed functional images were also checked individually for inclusion of the whole cerebrum and of the cerebellum. For rs‐fMRI data, head motion from volume to volume for each participant was computed using the mean framewise displacement.^39^, ^40^ Head motion was considered following the recommendation of Power *et al*.^40^ and described thoroughly in the Supplementary Methods.

### iCAPs and extraction of iCAPs activation measures

#### iCAPs

The iCAP analysis is an advanced tool for rs‐fMRI that identifies moments of significantly changing brain activity to extract large‐scale brain networks and their dynamic properties.^33^ We tailored the openly available code (https://c4science.ch/source/iCAPs/) in MATLAB R2016a to apply the iCAPs framework in VPT young adolescents including both before and after MBI rs‐fMRI. In short, total activation applies a voxel‐wise hemodynamically informed deconvolution^32^, ^41^ to the fMRI time series. Significant activation change points (i.e. transients), derived from deconvolved time series, were concatenated across all subjects in Montreal Neurological Institute (MNI) Average Brain space *via* a study‐specific DARTEL template previously created. Transients were then input into temporal k‐means clustering to derive simultaneously transitioning brain patterns, i.e. iCAPs. The optimum number of clusters, set to eight, was determined through consensus clustering (Supplementary Figs. S2 and S3). Time courses for all iCAPs were generated using spatiotemporal transient‐informed regression.^42^

#### iCAPs' labelling and temporal properties

For labelling iCAPs, reference was made to previous rs‐fMRI studies that utilized the iCAPs framework.^43^, ^44^, ^45^, ^46^ For each participant before MBI and after MBI, we obtained iCAPs' temporal characteristics by back projecting each iCAP into participants' activity‐inducing signals before and after MBI: (i) the total duration: total duration of overall activation as a percentage of the total nonmotion scanning time, a metric describing the amount of sustained activity (or functional ‘engagement’) for that specific iCAP; (ii) the occurrences: the number of activation blocks for each pair of iCAPs; (iii) the coupling: same‐signed coactivation; and (iv) the anticoupling: opposite‐signed coactivation. Couplings and anticouplings were calculated using the Jaccard score, i.e percent of joint activation time of two respective iCAPs reflecting the amount of overlap between the activity, in the same or the opposite signs, respectively, of each pair of iCAPs.

#### Static properties of inter‐iCAPs interactions

As supplementary analyses, a static measure of inter‐iCAPs interactions was calculated and compared before and after MBI for the eight identified networks (Supplementary Methods and Supplementary Table S9).

### Statistical analyses

Statistical analyses were completed using R version 4.0.3,^47^ RStudio version 1.3.1093,^48^ and MATLAB R2019b. The following steps were completed for statistical analyses:

1. Comparison of the VPT and FT groups on neurobehavioral measures

Comparisons between the VPT and the FT groups were performed using linear regressions for all neurobehavioral measures. Each neurobehavioral measure was modeled as the outcome variable, with the group serving as a fixed effect. *P*‐values were corrected for multiple comparisons with the false discovery rate (*q*‐values <0.1^49^). Secondary analyses were conducted to evaluate the impact of general intellectual abilities on outcome measures using the same linear regressions and adding the General Ability Index (GAI) as covariate.

2 Assessment of the effect of MBI on neurobehavioral measures significantly affected in the VPT group

The effect of MBI on neurobehavioral measures, which exhibited significant reductions in VPT participants compared with FT participants, was assessed using linear regressions. We modeled the neurobehavioral measures of interest as the outcome variables, the group as a fixed effect, and the subject as a random effect. *P*‐values were corrected for multiple comparisons with false discovery rate (*q*‐values <0.1^49^). Secondary analyses were conducted to evaluate group difference between the VPT group after MBI and the FT group on neurobehavioral measures.

3 Association between reliable changes in neurobehavioral outcomes and reliable changes in temporal properties of iCAPs (i.e. before and after the MBI)

- Calculation of the reliable change index (RCI)

For each neurobehavioral measure and each temporal property of iCAPs (i.e. duration, coupling, and anticoupling), the RCI was calculated for each participant.^50^ The RCI is a commonly applied approach to quantify a clinically significant change. It allows assessment of whether a patient's change over time on a given outcome measure cannot be attributable to error. A negative RCI signifies a decrease in scores between two time points, while a positive RCI indicates an increase in scores between two time points. The formula for the RCI is: RCI_score_ = *d*_i_/SEM_*d*_; where *d*_i_ is an individual‐level difference score (i.e. after MBI − before MBI) and SEM_*d*_ is the standard error of measurement, i.e. of the difference between the two test scores.

b Partial least‐square correlation

Partial least‐square correlation analyses (PLSC) were performed to evaluate multivariate patterns of correlation between: (i) clinical measures, including RCI in neurobehavioral measures as well as gestational age at birth and age at testing; and (ii) RCI in iCAPs' temporal properties. PLSC, a data‐driven multivariate technique, enhances the covariance between two matrices by identifying latent components (LCs) that represent linear combinations of the two matrices.^43^, ^51^, ^52^ To implement this, a publicly accessible MATLAB PLSC implementation was used: https://github.com/danizoeller/myPLS ^43^, ^52^ (see Supplementary Methods for detailed description of the PLSC applied).

In an initial PLSC, we investigated associations between clinical measures (32 × 7 matrix [*X*]) and reliable changes in the total duration of each iCAP (32 × 8 matrix [*Y*]). Subsequently, in a second PLSC, we delved into the relationships between clinical measures (*X*) and reliable changes in the occurrences of each pair of iCAPs (*Y*). In a third and fourth PLSC, associations between clinical measures (32 × 7 matrix denoted *X*) and reliable changes in the coupling and anticoupling of each pair of iCAPs (32 × 28 matrix denoted *Y*) were explored.

## Results

### Participant characteristics

The final sample included 32 VPT and 24 FT young adolescents aged between 10 to 14 years. Neonatal and demographic characteristics can be found in Table 2. In this final sample, parents reported neurodevelopmental disorders in 12.5% of young adolescents (*n* = 4), including 9.4% with dyslexia (*n* = 3) and 3.1% with dyspraxia (*n* = 1).

**Table 2:** Neonatal and demographic characteristics of VPT and FT participants

Characteristics	VPT (*n* = 32)	FT (*n* = 24)	Group comparison
*Neonatal characteristics*			
Gestational age, weeks (mean [SD])	29.07 (1.99)	39.89 (1.54)	*t*(54) = −22.153, *P* < 0.001
Birth weight, grams (mean [SD])	1197.66 (397.40)	3443.96 (452.92)	*t*(54) = −19.715, *P* < 0.001
IVH grades III and IV, *n* (%)	1 (3.1%)	0 (0%)	
cPVL, *n* (%)	1 (3.1%)	0 (0%)	
BPD, *n* (%)	8 (25%)	0 (0%)	
*Demographic characteristics*			
Age at assessment, months (mean [SD])	145.81 (14.83)	142.38 (12.85)	*t*(54) = 0.908, *P* = 0.368
Sex			
Female, *n* (%)	18 (56.3%)	9 (37.5%)	*X* ^2^(1) = 1.931, *P* = 0.165
Male, *n* (%)	14 (43.8%)	15 (62.5%)	
SES, Largo score (mean [SD])	3.91 (2.32)	3.05 (1.43)	*t*(52) = 1.5478, *P* = 0.128
GAI (mean [SD])	108.12 (10.55)	116.46 (11.04)	*t*(54) = −2.867, *P* = 0.006

In very preterm (VPT) young adolescents, age at assessment corresponds to the age before the mindfulness‐based intervention assessment. Socioeconomic status (SES) of the parents was estimated using the Largo scale.^63^ Higher Largo scores reflect lower SES of the parents. The Wechsler Intelligence Scale for Children, 5th Edition (WISC‐V^37^), was used to evaluate the General Ability Index (GAI) as a measure of general intellectual functioning (*M* = 100, SD = 15).

BPD, bronchopulmonary dysplasia; cPVL, cystic periventricular leukomalacia; FT, full‐term; IVH, intraventricular hemorrhage.

### Comparison between the VPT and FT groups on neurobehavioral measures

**Fig. 1:**
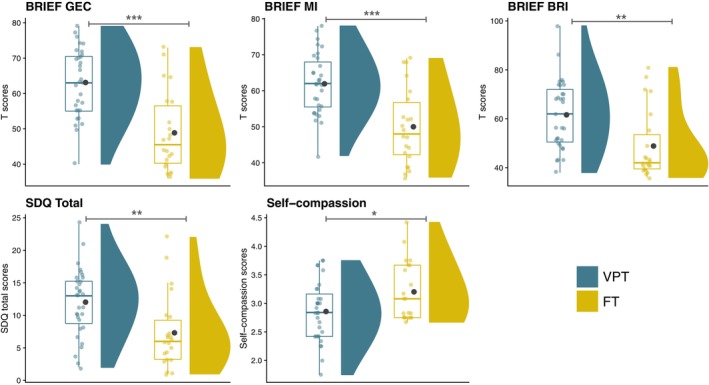
Neurobehavioral measures indicating significant group differences between the very preterm (VPT; before the mindfulness‐based intervention) and the full‐term (FT) groups using linear regression models (false discovery rate, *q*‐values <0.1). Dark blue = VPT; yellow = FT; group means are represented by a black dot; ***q < 0.001, **q < 0.01, *q < 0.1. BRIEF GEC, Behavior Rating Inventory of Executive Function, parent version (BRIEF) for the Global Executive Composite; BRIEF BRI, Behavioral Regulation Index; BRIEF MI, Metacognition Index; SDQ, Strengths and Difficulties Questionnaire, parent version.

The VPT group showed higher scores on the Behavior Rating Inventory of Executive Function, parent version (BRIEF) for the Global Executive Composite (BRIEF GEC; *q* < 0.001), Metacognition index (BRIEF MI; *q* < 0.001), Behavioral Regulation Index (BRIEF BRI; *q* < 0.01), and on the total score of the Strengths and Difficulties Questionnaire, parent version (SDQ total score; *q* < 0.01), reflecting increased executive and behavioral difficulties in daily life compared with the FT group. Moreover, the self‐compassion score was significantly reduced in the VPT group compared with the FT group (*q* < 0.1) (Figure 1). Other neurobehavioral measures were comparable between the VPT and FT groups (Supplementary Tables S3 and S4). Secondary analyses including the GAI as covariate showed comparable results (Supplementary Table S4).

### Effect of MBI on neurobehavioral measures significantly affected by preterm birth

**Fig. 2:**
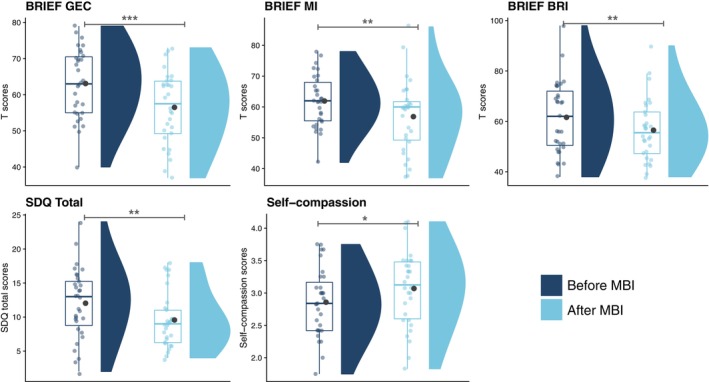
Neurobehavioral measures showing significant differences for the very preterm (VPT) group between before the mindfulness‐based intervention (MBI) and after the MBI using linear regression models (false discovery rate, *q*‐values <0.1). Dark blue = before MBI, light blue = after MBI. Means for before MBI and after MBI are represented by a black dot; ***q < 0.001, **q < 0.01, *q < 0.1. BRIEF GEC, Behavior Rating Inventory of Executive Function, parent version (BRIEF) for the Global Executive Composite; BRIEF BRI, Behavioral Regulation Index; BRIEF MI, Metacognition Index.

For measures showing significant differences between VPT and FT, significant score reductions were noted after MBI in the following: BRIEF GEC (*q* < 0.001), BRIEF MI (*q* < 0.01), BRIEF BRI (*q* < 0.01), and SDQ total score (*q* < 0.01). This indicates significantly less executive and behavioral difficulties in daily life following MBI. There was also a significant difference between self‐compassion scores before and after MBI, with increased self‐compassion scores after MBI in young VPT adolescents (*q* < 0.1) (Figure 2 and Supplementary Table S5). Secondary analyses comparing the VPT group after MBI and FT young adolescents showed no significant group difference that survived multiple comparison correction (Supplementary Table S6).

### Extracted iCAPs

**Fig. 3:**
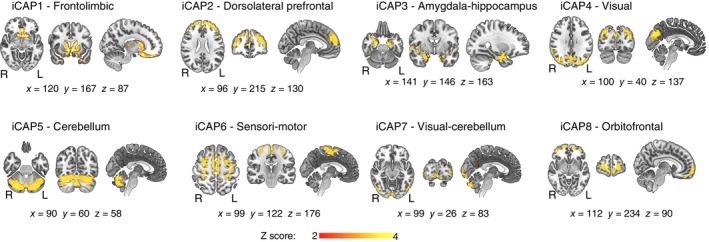
Spatial patterns of the eight innovation‐driven coactivation patterns (iCAPs) retrieved in very preterm (VPT) young adolescents before and after the mindfulness‐based intervention (MBI). Locations denote displayed slices in MNI Average Brain coordinates.

The iCAPs framework was applied to rs‐fMRI scans in VPT participants both before and after MBI. The eight extracted spatial maps represented in each iCAP align with recognized resting‐state networks and resemble commonly observed task‐related and cognitive networks in fMRI studies.^4^ These patterns also mirrored iCAP networks identified in earlier studies involving children, adolescents, and adults.^43^, ^44^, ^45^, ^46^ Specifically, the eight obtained networks (Figure 3), included: (i) sensory‐related networks, i.e. sensorimotor (iCAP6), visual (iCAP4), and visual‐cerebellum (iCAP7) networks; (ii) higher level cognitive networks, i.e. frontolimbic (iCAP1), dorsolateral prefrontal (iCAP2), and orbitofrontal (iCAP8) networks; and (iii) subcortical networks, i.e. amygdala‐hippocampus (iCAP3) and cerebellum (iCAP5) networks. Supplementary Table S7 shows in detail regions involved in each of the iCAPs networks.

### Association between reliable changes in neurobehavioral outcomes and reliable changes in temporal properties of iCAPs (i.e., before and after the MBI)

Only the first PLSC analysis applied on clinical measures (i.e., RCI in neurobehavioral measures as well as gestational age and age at testing) and reliable changes in the total duration of each iCAP gave a significant LC: LC 1 (*P* = 0.012). The three other PLSC applied on clinical measures and reliable changes in occurrences of each iCAP and in coupling and anticoupling of each pair of iCAPs did not show any significant associations. To reiterate, a negative RCI signifies a decrease in scores between the two time points, while a positive RCI indicates an increase in scores between them.

**Fig. 4:**
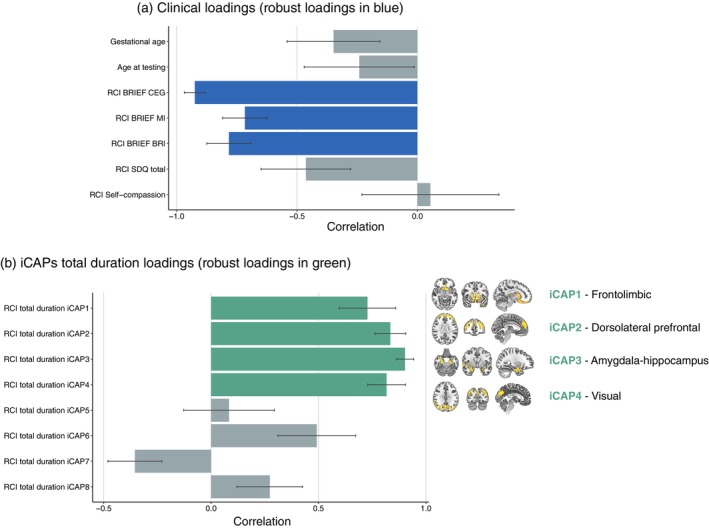
Associations between clinical measures and reliable changes (reliable change index [RCI] between before the mindfulness‐based intervention [MBI] and after the MBI) in the total duration of each innovation‐driven coactivation pattern (iCAP) based on the partial least‐square correlation analyses. (a) Clinical loadings: the diverging graph shows mean correlation averaged across bootstrap samples and their bootstrap‐estimated SDs (*x*‐axis) for each clinical measure (*y*‐axis); robust loadings are represented in blue. (b) iCAPs total duration loadings: the diverging graph shows mean correlation averaged across bootstrap samples and their bootstrap‐estimated SDs (*x*‐axis) for the reliable changes in the total duration of each iCAP (*y*‐axis); robust loadings are represented in green. For interpretation, a negative RCI signifies a decrease in scores between the two time points, while a positive RCI indicates an increase in scores between them.

Latent component 1 (*P* = 0.012) was observed in the context of the association between clinical measures (i.e., RCI BRIEF GEC, RCI BRIEF MI, RCI BRIEF BRI, RCI SDQ total, RCI Self‐compassion, gestational age, and age at assessment before MBI) and reliable changes in the total duration of each iCAP. Bootstrap‐estimated mean saliences, along with their bootstrap‐estimated SDs for clinical measures and reliable changes in the total duration of each iCAP, are detailed in Supplementary Table S8. Latent component 1 displayed a broad pattern of association between decreased RCI in BRIEF GEC, RCI BRIEF MI, and RCI BRIEF BRI with increased RCI in the total duration of iCAP1, iCAP2, iCAP3, and iCAP4, as illustrated in Figure 4a and 4b. In other words, a reduction in scores of the BRIEF after MBI, reflecting better executive abilities in daily life, was associated with an increase in the total duration of the frontolimbic (iCAP1), dorsolateral prefrontal (iCAP2), amygdala‐hippocampus (iCAP3), and visual (iCAP4) networks.

## Discussion

This study explored the benefit of an 8‐week MBI on executive, behavioral, and socioemotional abilities in VPT young adolescents and its association with reliable changes in the temporal dynamics of large‐scale brain networks. VPT young adolescents showed specific executive, behavioral, and socioemotional difficulties in everyday life compared with FT controls; these difficulties were significantly reduced after MBI in the VPT group. Our findings show that the enhancement observed in executive abilities after MBI, previously observed in a gold‐standard randomized controlled trial, is associated with reliable changes in temporal characteristics of large‐scale brain networks. More specifically, increased executive functioning in everyday life was associated with: (i) longer activation in frontolimbic and amygdala‐hippocampus networks known to be related with self‐regulation; (ii) longer activation in the dorsolateral prefrontal network known to be associated with attentional control; and (iii) longer activation in visual networks that have been related to attentional awareness of relevant sensory stimuli.

In the current sample, VPT young adolescents exhibited significant executive, behavioral, and socioemotional difficulties in everyday life in comparison to FT peers, as measured by parent‐reported and self‐reported questionnaires. The vulnerability of VPT children and adolescents for executive, behavioral, and socioemotional abilities has been well‐described in previous studies, and corresponds to the ‘preterm behavioral phenotype’ proposed in the literature.^2^

After an 8‐week MBI specifically designed for this age range, i.e. 10 to 14 years, we observed a significant reduction of executive, behavioral, and socioemotional difficulties in everyday life as well as a significant increase in self‐compassion. Furthermore, the significant difficulties in executive and socioemotional measures observed before the MBI in the VPT group compared with FT young adolescents no longer showed group differences after MBI. These findings are in line with the randomized controlled trial completed in a larger cohort of VPT young adolescents.^28^ This suggests that the improvement observed in executive, behavioral, and socioemotional measures following MBI in our study population is likely to be unrelated to a test–retest effect and is not only specific to the current sample of VPT young adolescents. Moreover, the beneficial impact of MBI on these abilities is consistent with previous studies conducted not only in typically developing adolescents but also in different clinical populations.^20^, ^21^

Considering the beneficial impact of MBI on vulnerable abilities in VPT young adolescents, we explored potential associations of behavioral benefits with temporal dynamics of large‐scale brain networks. For this purpose, a whole‐brain data‐driven approach was employed to unravel the transient activity of coordinated brain networks from spontaneous rs‐fMRI measurements. This approach aimed to extract dynamic features of FC across the entire brain.^33^ The findings showed a significant association between the improvement in executive abilities in everyday life after MBI with a longer activation of frontolimbic, dorsolateral prefrontal, amygdala‐hippocampus, and visual networks. These findings are coherent with previous studies conducted in adolescents as well as in adults both in healthy and clinical populations. Mindfulness‐mediated FC changes have been largely observed in frontolimbic as well as amygdala‐hippocampus networks.^53^, ^54^ These changes have been related to behavioral self‐regulation, including emotion regulation, as well as in the monitoring of social cognition.^22^, ^55^ Hippocampal activity has also largely been associated with mindfulness meditation and meditation expertise.^55^, ^56^ Also in line with previous studies, we observed an association between the improvement in executive functioning with a longer activation of the dorsolateral prefrontal network after MBI.^24^, ^53^ The changes in the recruitment of the dorsolateral prefrontal network have been associated with attentional control and the online processing of events and action.^22^ Increased activity within this region, as part of the executive network involved in controlling attentional resources to deal with immediate or future demands, has been associated with greater meditation experience.^57^ Additionally, the results showed that executive enhancement was associated with longer activation of the visual network involving the cuneus and precuneus, as well as superior and middle occipital regions. Mindfulness‐mediated FC changes in the visual networks have been consistently observed in the literature.^22^, ^24^, ^53^ Previous studies suggest that changes in visual connectivity could play a role in internally directed attention.^58^, ^59^ Within this concept, it has been hypothesized that mindfulness‐related processes could allow an increase in attentional awareness of relevant sensory stimuli normally suppressed.^22^, ^60^ Another proposed hypothesis is linked to the role of visual networks in a relaxation effect following mindfulness meditation as it has been shown that FC of the visual cortex is increased during light sleep, sedation, and alcohol consumption.^55^, ^61^ Finally, there was no association between the gain observed after MBI on any of the neurobehavioral measures with reliable changes in the occurrence, nor in the coupling or anticoupling of the different networks extracted.

A recent study on the same cohort exploring white‐matter microstructural changes associated with neurobehavioral changes after MBI corroborate these findings.^62^ This prior study demonstrated that enhanced executive functions in daily life were associated with increased structural connectivity using a tractography‐based approach characterized by increased fractional anisotropy and reduced axonal dispersion. Remarkably, this previous study exhibited high regional overlaps with the current study, reflected by changes both in the microstructural properties of white‐matter tracts and in dynamic FC. Specifically, longer activation in the frontolimbic and amygdala‐hippocampus networks might correspond to increased structural connectivity in the cingula, anterior thalamic radiations, and other thalamic tracts observed in the prior study. Longer activation in the dorsolateral prefrontal network might coincide to increase in structural connectivity in the genu of the corpus callosum as well as in other tracts involving the dorsolateral prefrontal cortex (e.g., arcuate fascicles and inferior occipitofrontal fascicule). Finally, longer activation in visual networks might correspond to increase structural connectivity in the optic radiations and other occipital tracts. Altogether, enhancements in everyday executive functioning following MBI appear to be associated with concurrent structural and functional changes in overlapping brain regions.

Of note, the presence of neurodevelopmental disorders, specifically dyslexia and dyspraxia, within our sample may not exert a substantial influence on our findings for several compelling reasons. First, the analyses are centered on examining intraindividual changes rather than making direct comparisons with an external group. Second, we would expect MBI to have an impact on attention‐deficit/hyperactivity disorder, which was not reported in our current sample.

Our study has limitations that require mentioning. Although embedded within a broader randomized controlled trial,^28^, ^35^ this study employed a pre–post intervention design exclusively within the VPT group, which completed the MBI as the MRI scan was only completed before and after MBI. Consequently, a notable limitation of this study is the absence of an active control condition or a placebo condition. It is imperative to underscore that comparing the effects of the MBI against an engaging active control condition is indispensable for ensuring a robust evaluation of the impact on neurobehavioral measures and the related changes in FC dynamics among VPT young adolescents. Second, the group difference observed as well as the beneficial effect of MBI was only observed *via* self‐ and parent‐reported questionnaires. The subjective aspect of these tools is well‐documented.^63^ The completion of questionnaires by multiple informants from different settings (e.g., parents and teachers) could be considered in future studies to give a more objective view of the changes occurring after MBI.^64^, ^65^ In addition, it is possible that neurobehavioral tests employed in this study may not have effectively captured the everyday life challenges faced by these young adolescents.^66^ While our findings are promising, a well‐designed active control condition or placebo condition are needed to further investigate the neural signature of positive clinical changes after MBI. Moreover, in the context of a mindfulness study, specifically targeting a given mindful practice during a functional MRI sequence (e.g. grounding meditation)^35^ and instructing participants accordingly could bring further insight into the underlying process of a specific practice with neurobiological underpinning and associated neurobehavioral changes.^67^

## Disclosure statement

The authors have no competing interests to declare that are relevant to the content of this article.

## Author contributions

V.S.: conceptualization; data curation; formal analysis; investigation; methodology; project administration; software; visualization; writing – original draft; writing – review & editing. M.C.L.: conceptualization; data curation; investigation; methodology; project administration; writing – review & editing. N.F.: writing – review & editing. L.G.A.F.: data curation; Investigation; writing – review & editing. C.B.T.: conceptualization; investigation; project administration; resources; supervision; validation; writing – review & editing. D.V.D.V.: methodology; resources; software; supervision; writing – review & editing. P.S.H.: conceptualization; funding acquisition; methodology; project administration; resources; supervision; validation; writing – review & editing. R.H.V.L.: conceptualization; investigation; methodology; project administration; resources; supervision; validation; writing – review & editing.

## Funding information

The authors have no relevant financial or nonfinancial interests to disclose.

## Ethics statement

All experimental protocols were approved by the Swiss ethics committees on research involving humans, ID: 2015‐00175. All methods were performed in accordance with relevant guidelines and regulations. Written informed consent was obtained from primary caregivers and participants.

## Supporting information


**Supplementary Table S1.** Theme addressed during each session of the MBI.
**Supplementary Table S2.** Details of the neurobehavioral outcome measures and scores.
**Supplementary Table S3.** Group comparison on neurobehavioral outcome measures of the VPT before MBI and FT young adolescents. Linear regression models included each neurobehavioral measures as outcome variable and the group as a fixed effect (i.e. VPT [before MBI] and FT).
**Supplementary Table S4.** Group comparison on neurobehavioral outcome measures of the VPT and FT young adolescents, including the General Ability Index (GAI) as covariate. Linear regression models included each neurobehavioral measures as outcome variable and the group as a fixed effect (i.e. VPT and FT) and GAI as covariate.
**Supplementary Table S5.** Comparison of neurobehavioral scores before and after MBI intervention in VPT young adolescents. Linear regression models included each neurobehavioral measures that showed a significant difference before and after MBI as outcome variable, before/after MBI as a fixed effect, and subject as a random effect.
**Supplementary Table S6.** Group comparison on neurobehavioral outcome measures of the VPT after MBI and FT young adolescents. Linear regression models included each neurobehavioral measures as outcome variable and the group as a fixed effect (i.e. VPT after MBI and FT).
**Supplementary Table S7.** iCAPs functional networks of regions from the automated anatomical labelling 2 (AAL2) atlas. Percentiles indicate the fraction of voxels of a functional network or region that have a *z*‐score >2. A network/region is listed if more than 20% of the network/region is included in the iCAP.
**Supplementary Table S8.** Bootstrapping means and SDs of the loadings for clinical and reliable change in the total duration of each iCAP of the PLSC analyses.
**Supplementary Table S9.** Comparison of before and after MBI in VPT participants for static inter‐iCAPs correlations. Linear regression models showed no significant difference in static functional connectivity before and after MBI.
**Supplementary Figure S1.** Participant flow chart. All neuropsychological assessments and magnetic resonance imaging (MRI) acquisitions were completed at the Campus Biotech in Geneva, Switzerland. MBI, Mindfulness‐based intervention.
**Supplementary Figure S2.** Consensus clustering quality measures for cluster numbers *K* = 5 to *K* = 15. The distribution of average consensus values per cluster: the mean for each cluster is represented by a black line and the median is represented by a gray line. Mean consensus is maximal (closest to 1) for *K* = 8.
**Supplementary Figure S3.** Consensus matrices for cluster numbers *K* = 5 to *K* = 15. High values in the matrix indicate that the two corresponding frames were clustered together during re‐sampling. A value of 1 means that the frames were always clustered together, while a value of 0 means that the frames were never clustered together. We selected to use *K* = 8 based on visual inspection of the consensus matrices and evaluation of consensus clustering quality measures (Monti *et al*., 2003). In Supplementary Fig. S1, we plot the distribution of average consensus values per cluster to assess the quality of the clustering for each *K*.

## Data Availability

Ethical restriction prevent us from making anonymised data available in a public repository. Deidentified individual participant data (including data dictionaries) will be made available, in addition to study protocols to researchers who provide a methodologically sound proposal for use in achieving the goals of the approved proposal. Proposals should be submitted to Russia.HaVinhLeuchter@unige.ch.
