# Supplementary material for: Impact of a mindfulness‐based intervention on neurobehavioral functioning and its association with large‐scale brain networks in preterm young adolescents
Source: Psychiatry Clin Neurosci. 2024 May 17;78(7):416–25. doi: 10.1111/pcn.13675 (PMC11488620; doi:10.1111/pcn.13675)
Supplement: Supplementary file 1 — Supplementary Table S1. Theme addressed during each session of the MBI. Supplementary Table S2. Details of the neurobehavioral outcome measures and scores. Supplementary Table S3. Group comparison on neurobehavioral outcome measures of the VPT before MBI and FT young adolescents. Linear regression models included each neurobehavioral measures as outcome variable and the group as a fixed effect (i.e. VPT [before MBI] and FT). Supplementary Table S4. Group comparison on neurobehavioral outcome measures of the VPT and FT young adolescents, including the General Ability Index (GAI) as covariate. Linear regression models included each neurobehavioral measures as outcome variable and the group as a fixed effect (i.e. VPT and FT) and GAI as covariate. Supplementary Table S5. Comparison of neurobehavioral scores before and after MBI intervention in VPT young adolescents. Linear regression models included each neurobehavioral measures that showed a significant difference before and after MBI as outcome variable, before/after MBI as a fixed effect, and subject as a random effect. Supplementary Table S6. Group comparison on neurobehavioral outcome measures of the VPT after MBI and FT young adolescents. Linear regression models included each neurobehavioral measures as outcome variable and the group as a fixed effect (i.e. VPT after MBI and FT). Supplementary Table S7. iCAPs functional networks of regions from the automated anatomical labelling 2 (AAL2) atlas. Percentiles indicate the fraction of voxels of a functional network or region that have a z‐score >2. A network/region is listed if more than 20% of the network/region is included in the iCAP. Supplementary Table S8. Bootstrapping means and SDs of the loadings for clinical and reliable change in the total duration of each iCAP of the PLSC analyses. Supplementary Table S9. Comparison of before and after MBI in VPT participants for static inter‐iCAPs correlations. Linear regression models showed no significant difference in [file PCN-78-416-s001.pdf]

Supplementary Materials

Supplementary Methods

Mindfulness-based intervention

The proposed MBI was designed by the authors, adapting well-known MBI programs including Mindfulness-Based Stress Reduction (MBSR; [1]) and Mindfulness-Based Cognitive Therapy (MBCT; [2]) to adolescents' needs and language. The length of sessions and practices were also adapted to this population. The program consisted of 8 weekly sessions in groups of up to 8 participants, lasting 1h30. Two MBI groups were offered per week (Wednesdays and Fridays) and participants had the possibility to choose the most convenient day for them. Two MBI instructors were present for each group throughout the intervention.

For each session one theme was addressed, such as attention and the stabilisation of the focus of attention, bodily sensations, breath, emotions, thoughts, compassion, stress, stress reactivity and coping strategies. Different formal meditation practices were introduced, such as sitting mediation with different objects of attention, body scan, walking meditation and mindful movement. Participants had the opportunity to share experiences with their peers and the instructors during each session, allowing the recognition of individual patterns of behaviour, and at the same time, the appreciation of the shared experiences as a community. Compared to the MBSR and MBCT programs, all formal practices were shorter. We also added playful practices such as a card game needing very quick responses to win the game; with the objective of helping young adolescents to become aware of their bodily reactions to emotions. They were also invited to practice between 5 to 20 minutes per day at home, using guided mediation recorded by the instructors. The details about each session can be visualised in Supplementary Table S1.

Magnetic Resonance Imaging (MRI) acquisition and preprocessing

Magnetic Resonance Imaging acquisition

MRI data were acquired at the Campus Biotech in Geneva, Switzerland, using a Siemens 3T Magnetom Prisma scanner. All participants completed a simulated "mock" MRI session prior to their first MRI scan. This preparation process was conducted by trained research staff and allowed participants to familiarise themselves with the scanner and the scanning process, eventually raising any concerns they might have had prior to the MRI scan. Furthermore, this process is known to facilitated acquisition of good quality MRI images in paediatric population [3, 4]. Structural T1-weighted MP-RAGE (magnetization-prepared rapid gradient-echo) sequences was acquired using the following parameters: voxel size = 0.9 × 0.9 × 0.9 mm3; repetition time (TR) = 2,300 ms; echo time (TE) = 2.32 ms; inversion time (TI) = 900 ms; flip angle (FA) = 8°; and field of view (Fov) = 240 mm. Resting-state functional images were T2*-weighted with a multislice gradient-echo-planar imaging (EPI) sequence of 64 slices; voxel size = 2 x 2 x 2 mm3; TR = 720 ms; TE = 33 ms; Fov = 208 mm; 500 frames; acquisition time = 6 minutes. During the rs-fMRI sequence, young adolescents were asked to keep their eyes closed and engage in mind wandering. Finally, a fieldmap was acquired each time a participant entered the scanner, with TR = 627 ms; TE1 = 5.19 ms; TE2 = 7.65 ms; and FA = 60°.

Resting-State functional MRI data preprocessing

Our data were preprocessed using SPM12 (Wellcome Department of Imaging Neuroscience, UCL, UK) in MATLAB R2016a (The MathWorks, Inc., Natick, Massachusetts, United States). Rs-fMRI data were converted from the native DICOM to NIFTI format and the preprocessing pipeline described by Freitas, Liverani and colleagues was used.[5, 6] Rs-fMRI images were spatially realigned and unwarped using the fieldmap images acquired, respectively, to correct for motion artefacts and potential geometric distortions. The unwarping step allows to improve the co-registration between structural and functional images and to reduce the distortion variability across subjects during spatial normalisation to a common space.[7] Functional images were then coregistered to their corresponding structural images in subject space. Structural images were segmented with the SPM12 segmentation algorithm to automatically identify different tissue types within the images, i.e., grey matter, white matter, cerebrospinal fluid;[8] and a study-specific template was generated using Diffeomorphic Anatomical Registration using Exponential Lie algebra (DARTEL [9]) that will be used in the Innovation-Driven Co-Activation Patterns (iCAPs) framework described below. Finally, the first five rs-fMRI images were excluded. For each participant, preprocessed functional images were also check individually for inclusion of the whole cerebrum and of the cerebellum.

Head motion

For rs-fMRI data, the mean framewise displacement for each frame was computed to quantify the extent of head motion from volume to volume for each participant.[10, 11] Following the recommendation of Power and colleagues (2014), for a frame with a mean framewise displacement above the threshold of 0.5 mm, we also considered

one frame before and two after. If more than 20% of the frames were above threshold (including one frame before and two after), the participant was removed from further analyses. Based on these criteria, 7 VPT participants were excluded from further analyses.

Considering all frames (including the one removed following the recommendation of Power and colleagues (2014)) in the remaining 32 VPT participants, paired-sample t-test was used to assess difference in framewise displacement between before and after MBI. The paired-sample t-test showed no significant different between before and after MBI ($t(31) = 1.96$, $p = 0.06$). Moreover, association between detected innovation (i.e., transients) and framewise displacement was assessed by computing, for each subject, the mean correlations between the framewise displacement matrix and the innovation matrix (i.e., transients map). The mean correlation for all VPT participants before MBI was r= -0.0069, ranging from r = -0-070 to r = 0.0671. The mean correlation for all VPT participants after MBI was r= -0.0073, ranging from r = -0-0655 to r = 0.0657. The very weak correlations illustrate the lack of association between the extracted transients used to establish the iCAPs networks and head movement.

Innovation-Driven Co-Activation Patterns (iCAPs) and extraction of iCAPs activation measures

Innovation-Driven Co-Activation Patterns (iCAPs)

Innovation-driven-co-activation-pattern (iCAP) analysis is a state-of-the-art rs-fMRI analysis tool that allows to derive a set of whole-brain spatial patterns of regions whose activity simultaneously increases or decreases, thus characterised by similar functional dynamic behaviour.[12] For a comprehensive explanation of the methodology and procedure, we refer to Karahanoglu and colleagues (2015) and to Zöller and colleague (2019). We tailored the openly available MATLAB code (https://c4science.ch/source/iCAPs/) on MATLAB vR2016a (The MathWorks, Inc., Natick, MA) to apply the iCAPs framework in VPT young adolescents including both before MBI and after MBI rs-fMRI. The overall routine is composed of 4 steps:

- Total activation (TA): First, TA applies a voxel-wise hemodynamically-informed deconvolution [14, 15] to the fMRI timeseries in a way that promotes the temporal sparsity of activity transients and spatially coherent activations. TA provides three types of information: (1) activity-related signals that are denoised fMRI signals; (2) sustained, or block-type, activity-inducing signals that are deconvolved signals; (3) innovation signals that are the derivative of the activity-inducing signals and encode transient brain activity episodes as spikes.
- Detection of significant transients: Innovation signals are computed as the temporal derivative of the deconvolved signals. The obtained signals can be seen as a representation in terms of transients in brain activity, where large amplitude transients implicitly identify change-points. Significant transients were determined using a two-step thresholding procedure. A temporal threshold estimated from a surrogate distribution, keeping only transients larger than 95% or lower than

5%. Then, a spatial thresholding procedure was applied, in which a frame was considered significant if at least 5% of the grey matter voxels were active. The frames showing significant transients are called innovation frames, and allow to identify time-points when a given region in the brain undergoes an increase or decrease in activity.

- Aggregation: The significant, i.e., innovation, frames were warped into MNI (Montreal Neurologic Institute) space via a study-specific DARTEL template previously created (see preprocessing). All frames were then aggregated for clustering.
- Temporal clustering: The retained innovation frames, underwent K-means clustering to label timepoints with consistent brain patterns of transitions, i.e., iCAPs. The optimum number of 8 clusters was determined by consensus clustering and following recommendation of previous studies (Supplementary Figure S1 and S2).[13, 15, 16]
- Time courses extraction: Time courses were obtained for all iCAPs using spatiotemporal transient-informed regression.[17]

iCAPs' labelling and temporal properties

To guide iCAPs labelling, previous studies rs-fMRI studies, including studies using the iCAPs framework were used as reference.[13, 18-20] For each participant before MBI and after MBI, we obtained iCAPs' temporal characteristics by back projecting each iCAP into participants' activity-inducing signals before and after MBI: (1) the total duration, i.e., the total duration of overall activation as percentage of the total non-motion scanning time, a metric describing the amount of sustained activity (or functional "engagement") for that specific iCAP; (2) the occurrences: the number of activation blocks; for each pair of iCAPs: (3) the coupling: same-signed co-activation, and (4) the anti-coupling: opposite-signed co-activation. Couplings and anti-couplings were calculated using the Jaccard score; i.e., percent of joint activation time of two respective iCAPs and reflect the amount of overlap between the activity, in the same or the opposite signs, respectively, of each iCAPs' pair.

Static properties of inter-iCAPs interactions

We extracted the activity time course for each iCAP by spatio-temporal back-projection of the spatial maps onto the activity-inducing signals.[17, 20] As a result, a set of signals was generated for each run describing activity segments of each of the iCAPs. Subsequently, a static measure of inter-iCAPs interactions was calculated corresponding to temporal correlation. The matrices representing the inter-iCAPs correlations for each participant were subsequently employed to for before and after MBI comparisons. Linear regression models were used to compare static inter-iCAPs correlations before and after MBI for the 8 identified networks. The results showed no group difference for all of the static inter-iCAPs correlations (Supplementary Table S9).

Statistical analyses

Association between reliable changes in neurobehavioural outcomes and reliable changes in temporal properties of iCAPs (i.e., before and after the MBI)

For each neurobehavioural measures, each iCAPs' temporal properties (i.e., duration, coupling and anti-coupling) and each participant, the reliable change index (RCI) was calculated.[21] The RCI is a commonly applied approach to quantify a clinically significant change. It allows to assesses whether a patient's change over time on a given outcome measure that cannot be attributable to error. Negative RCI indicates a reduction of the scores between two time points, whereas positive RCI indicates an increase in scores between two time points. The formula for the RCI is: $RCI_{score} = d_i / SEM_d$; where d_i is an individual-level difference score (i.e., after MBI – before MBI) and SEM_d is the standard error of measurement, i.e., of the difference between the 2 test scores.

Partial least square correlation analyses (PLSC) were performed to evaluate associations between: (i) clinical measures, including reliable changes in neurobehavioural measures as well as gestational age at birth and age at testing, and ;(ii) reliable changes in iCAPs' temporal properties. PLSC is a data-driven multivariate technique that maximizes the covariance between two matrices by identifying latent components (LCs) which are linear combinations of the two matrices.[22-24] A publicly available Matlab PLSC implementation was used: https://github.com/danizoeller/myPLS.[23, 24]

In a first PLSC, associations between clinical measures and reliable change in the total duration of each iCAPs were explored. The clinical measures refer to the five RCI of neurobehavioural measures showing significant difference between the VPT and FT group (i.e., RCI BRIEF GEC, RCI BRIEF MI, RCI BRIEF BRI, RCI SDQ total, RCI Self-compassion), as well as gestational age at birth and age at assessment before MBI. The clinical data were stored in a 32 x 7 matrix denoted X. Each row of X represents one subject and the matrix's 5 columns are made up of the five clinical scores (i.e., RCI BRIEF GEC, RCI BRIEF MI, RCI BRIEF BRI, RCI SDQ total, RCI Self-compassion, gestational age, age at assessment). Reliable change in the total duration of each iCAPs were gathered in a 32 x 8 matrix denoted Y, with each row matching one subject and each column matching the RCI of total duration of each the 8 iCAPs. A cross-covariance matrix was then computed between X (participants x clinical values) and Y (participants x reliable change in the total duration of each iCAPs). Singular value decomposition was then applied to this cross-

covariance matrix, resulting in latent components. Each latent component is composed of a set of clinical loadings and reliable change in the total duration of each iCAPs loadings, akin to structure coefficients. Structure coefficients lie between –1 and 1, and can be interpreted similarly to correlation values. Structure coefficients or loadings reflect the direct contribution of a specific predictor to the predictor criterion independently of others, which can be critical when predictors are highly correlated between each other (i.e., in presence of multicollinearity [25]). Here, loadings indicate how strongly each clinical measures and reliable change in the total duration of each iCAPs contribute to the multivariate association of clinical measures and reliable change in the duration of each iCAPs. The significance of latent components was determined by permutation testing (1000 permutations) and considered robust at $p<0.01$. Stability of clinical loadings and reliable change in the total duration of each iCAPs loadings were estimated using bootstrapping (500 bootstrap samples with replacement). The bootstrap ratio z-scores were obtained as normalized estimates of robustness for each clinical measure and each reliable change in the total duration of each iCAPs, by dividing each clinical and reliable change in the total duration of each iCAPs bootstrap mean loading by its bootstrap-estimated standard deviation. The bootstrapped ratio z-scores were converted to p-values. The contribution of clinical loadings and reliable change in the total duration of each iCAPs loadings for a given latent component was considered robust at $p < 0.001$ (i.e., absolute bootstrap ratio z-scores above 3 or below -3), which is approximately equivalent to a 99% confidence interval.[26]

In a second PLSC, associations between clinical measures and reliable change in the occurrences of each pair were explored. A procedure similar to the first PLSC was employed using a 32 x 7 matrix denoted X containing the clinical measures and a 32 x 8 matrix denoted Y containing the reliable change in the occurrences of iCAPs. In a third PLSC, associations between clinical measures and reliable change in the coupling of each pair of iCAPs were explored. A procedure similar to the first PLSC was employed using a 32 x 7 matrix denoted X containing the clinical measures and a 32 x 28 matrix denoted Y containing the reliable change in the coupling of each pair of iCAPs. In a fourth PLSC, associations between clinical measures and reliable change in the anti-coupling of each pair of iCAPs were explored. A procedure similar to the first PLSC was employed using a 32 x 7 matrix denoted X containing the clinical measures and a 32 x 28 matrix denoted Y containing the reliable change in the anti-coupling of each pair of iCAPs.

Supplementary Tables

Supplementary Table S1. Theme addressed during each session of the MBI

Session	SESSION THEME<br>Session Intention<br>Session Mindfulness Attitude	Agenda	Home practice
1	ATTENTION and AUTOPILOT		
	Introduction to attention and autopilot mode<br>Beginners Mind	Group and instructor introduction<br>Mindfulness definition: To pay attention to what happens in the present moment, with curiosity and non-judgmentally.<br>Dialogue about attention awareness and focus of attention<br>The 6 channels: 5 senses and thoughts<br>Practice: Eating a raisin as an explorer<br>Practice: Grounding mediation<br>Closure practice	Chart: Attention - where is my attention now?<br>Practice: Mindful eating – a mindful bite once a day<br>Practice: Grounding meditation
2	DISCOVERING THE BODY LANGUAGE		
	Discovering bodily sensations<br>Acceptance	Opening practice: Grounding meditation<br>Dialogue about home practice<br>Dialogue about sensations and sensation awareness<br>Practice: Lying down Body scan, with a component of contraction and relaxation at the beginning<br>Practice: Seated body scan<br>Closure practice	Chart: Cool moment of the day<br>Practice: Doing mindfully something habitually done on autopilot<br>Practice: Grounding meditation<br>Practice: Body scan
3	ATTENTION STABILIZATION		
	Discovering the breath<br>Non-striving	Opening practice: Grounding meditation<br>Dialogue about home practice<br>Dialogue about the breath and it's use as a possible anchor<br>Practice: The 3 minutes' break<br>Practice: Stop and breath<br>Practice: Yoga posture<br>Closure practice	Chart: Bad moment of the day<br>Practice: Body scan<br>Practice: 3 minutes' break<br>Practice: Stop and breath<br>Practice: Yoga posture
4	RECOGNIZING EMOTIONS		
	Recognizing emotions from bodily sensations	Opening practice: Grounding meditation<br>Dialogue about home practice<br>Practice: Seated body scan, including emotion<br>Dialogue about emotions: recognizing emotions from bodily sensation	Chart: Emotions - recognizing links between sensations, thoughts and behavior<br>Practice: Yoga posture<br>Practice: The 3 minutes' break<br>Practice: Sitting meditation (emotions)

		Practice: Sitting meditation: after remembering a difficult moment, recognize the emotion(s) and it'-s manifestations<br>Drawing and naming the identified emotion<br>Closure practice: The 3 minutes' break	
5	RECOGNIZING THOUGTHS		
	I'm much more than my thoughts<br>Non-judging	Opening practice: Grounding meditation<br>Dialogue about home practice<br>Walking down the street exercise<br>Discussion about thoughts and emotions<br>Practice: walking meditation<br>Closure practice	Chart: Cool moment of the day - noting sensations, thoughts and behavior<br>Practice: Sitting meditation (emotions)<br>Practice: Walking mediation
6	AUTOMATIC REACTION OR CONSCIOUS RESPONSE?		
	Exploring stressors and stress reaction<br>Letting go	Opening practice: Grounding meditation<br>Dialogue about home practice<br>Quick board game: identifying stress reaction<br>Discussion about stressors and stress reaction strategies<br>Practice: 5 minutes to deal with stress<br>Closure practice	Chart: Bad moment of the day - noting sensations, thoughts and behavior<br>Chart: Identifying qualities on others<br>Practice: 5 minutes to deal with stress<br>Practice: Walking mediation
7	KINDNESS		
	Being kind to oneself and to others<br>Gratitude and generosity	Opening practice: Grounding meditation<br>Dialogue about home practice<br>Discussion about kindness and compassion<br>Practice: finding refuge<br>Drawing our refuge<br>Mindful listening exercise<br>Dialogue about communications and social media<br>Closure practice	Letter to oneself:<br>What did I learned and I don't want to forget?<br>What did I learn about myself?<br>Which meditations do I want to keep practicing?<br>Practice: Refuge<br>Practice: Chose another meditation to practice
8	CLOSURE AND OPENNESS		
	Integrating the program<br>Trust	Opening practice: Grounding meditation<br>Dialogue about home practice<br>Practice: sitting meditation about the program<br>Satisfaction questionnaire<br>How to facilitate our own practice<br>Closing ritual	

Supplementary Table S2. Details of the neurobehavioural outcome measures and scores

Domains	Modalities	Measures	Description	Scores
Executive abilities				
	Parent questionnaire			
		Behaviour Rating Inventory of Executive Function, parent version (BRIEF, Gioia, Isquith [27])		
			The BRIEF parent questionnaire provides an index of attention, hyperactivity and impulsivity in everyday life. The BRIEF comprises 86 items over two standardised subscales: (i) Behavioural Regulation Index (BRI) comprising 3 subscores including, Inhibit, Shift, Emotional Control; (ii) Metacognition index (MI) comprising 5 subscores including, Initiate, Working Memory, Plan/Organise, Organisation of Materials, Monitor; as well as a global score called the Global Executive Composite (GEC). Higher scores reflect increased difficulties in executive functioning (M=50; SD=10).	BRIEF GEC<br>BRIEF BRI<br>BRIEF MI
	Neuropsychological tests			
		Letter-Number Sequencing (WISC-IV, Wechsler [28])		
			The letter-number sequencing is a working memory task. Sequences of number and letters are read to the participant, and he/she is then asked to re-sequence the numbers in numerical order from lowest to highest and then to sequence the letters in alphabetical order. Standardised scores were used (M=10; SD=3). Higher standardised scores reflect higher working memory skills.	Letter-number sequencing
		Tempo Test Rekenen [29]		
			The Tempo Test Rekenen is an arithmetic test consisting of 200 arithmetic number fact problems presented in five rows (one row with addition, one row with subtraction, one row with division, one row with multiplication, and one mixed problem row). Within each row, the problems increase in difficulty. Participant are asked to solve as many items as possible within 1 min per row. The total raw score was used. Higher total scores reflect higher arithmetic skills.	Tempo test
	Neurocognitive computerised tasks			
		Flanker Visual Filtering Task [30]		
			The Flanker Visual Filtering Task was used to assess attentional control and information processing speed. Each trial showed a horizontal row of five fish. The participant was asked to respond as quickly as possible to whether the central fish was facing to the left or right. Congruent trials were the ones with all five fish in the horizontal row pointing in the same direction and incongruent trials were the ones with the four distracting fishes pointing in the opposite direction of the central target	-Flanker processing speed<br>-Flanker inhibition

		fish. Mean reaction time of the congruent condition was used to assess information processing speed. Higher mean reaction times reflect slower processing speed. The inhibition score (reaction time in incongruent conditions – reaction time in congruent conditions) was used as a measure of attentional control. Higher inhibition scores reflect increased difficulties in attentional control.	
		Reality Filtering Task [31, 32]	
		The Reality Filtering task child-adapted version was used to assess recognition memory and orbitofrontal reality filtering. It consisted of a continuous recognition task composed of two runs with the same picture set but arranged in different order. The Temporal Context Confusion index was used as a reality filtering score (TCC as defined by Schnider, 2018): TCC = (FP2/Hits2) – (FP1/Hits1), where FP2 and FP1 are the false positives in run 2 and run 1 (i.e., the incorrect "yes" responses to images that have not yet been presented in the ongoing run), and Hits2 and Hits1 are the correct recognitions of targets in run 2 and run 1 respectively (i.e., the correctly-recognized repetitions). Higher TCC score indicate increased reality filtering difficulties.	Reality filtering TCC
Behaviour and socio-emotional abilities			
	Parent questionnaire		
		Strength and Difficulties Questionnaire, parent version (SDQ, Goodman [33])	
		The SDQ parent questionnaire assesses overall behaviour problems, emotional symptoms, hyperactivity and inattention, peer relationship problems, and prosocial behaviour. It rates participant's behaviour over the previous 6 months. The SDQ is scored on a Likert scale and includes 25 items, providing a Total Difficulties score. Higher Total Difficulty scores reflect increased behavioural and socio-emotional difficulties.	SDQ total
	Self-reported questionnaires		
		KIDSCREEN-27 [34]	
		The KIDSCREEN-27 is a self-reported questionnaire providing an index of health-related quality of life in children and adolescents. This instrument scored on a Likert scale and includes 27 items, providing a total score. Higher total scores reflect increased quality of life and well-being.	KIDSCREEN total
		Social Goal Scale (SGS, Patrick, Hicks and Ryan [35])	
		The SGS is a self-reported questionnaire providing an index of social responsiveness and of goals setting which ultimately gets you involve with some social work. This instrument scored on a Likert scale and includes 11 items providing a total score. Higher total scores reflect increased social goal in daily life.	Social goal
		Self-Compassion Scale – Short form (SCS, Raes, Pommier [36])	
		The SCS is a self-reported questionnaire comprising 12 items, which produces a total score. Higher total scores reflect increased self-compassion.	Self-compassion
	Neuropsychological tests		
		Affect Recognition (NEPSY-II, Korkman, Kirk and Kemp [37])	

		The affect recognition subtest assesses the ability to recognise facial emotional expressions (happy, sad, anger, fear, disgust, and neutral) from photographs of children’s faces in several matching tasks. In the first task, the participant selected one of the four faces that depicted the same emotion as a child's face at the top of the page. In a second task, the participant selected two photographs of faces that displayed the same affect from a selection of four photographs. Finally, the participant examined a photograph of a child's face for 5 seconds, and then from memory, selected two photographs that matched the same emotion as the face previously shown. Standardised scores were used (M=10; SD=3). Higher scores reflect better affect recognition skills.	Affect recognition
		Theory of Mind (NEPSY-II, Korkman, Kirk and Kemp [37])	
		The theory of mind subtest measures understanding of mental functions and other people's perspectives.<br>In the first task, questions are asked to the participant about different verbal scenarios measuring understanding of beliefs, intentions, others’ thoughts, ideas and comprehension of figurative language. In the second task, participants have to match facial emotional expressions, from photographs of children’s faces, to a scenario. The total raw score was age-adjusted for each participant using the procedure described in the main statistical analyses section. Higher scores reflect better theory of mind skills.	Theory of mind

Supplementary Table S3. Group comparison on neurobehavioural outcome measures of the VPT before MBI and FT young adolescents. Linear regression models included each neurobehavioural measures as outcome variable and the group as a fixed effect (i.e., VPT (before MBI) and FT).

	VPT (before MBI)		FT		Group comparisons
	Mean (SD)	Range	Mean (SD)	Range	
BRIEF CEG	63.1 (9.66)	40-79	48.86 (11.36)	36-73	R^2 = 0.32, F (1,51) = 24.131, p<0.001, q<0.001
BRIEF MI	61.94 (8.45)	42-78	50 (10.53)	36-69	R^2 = 0.29, F (1,51) = 20.915, p<0.001, q<0.001
BRIEF BRI	61.61 (14.21)	38-98	48.86 (14.12)	36-81	R^2 = 0.17, F (1,51) = 10.415, p=0.002, q=0.009
Letter-Number sequencing	10.56 (2.29)	5-15	11.26 (2.34)	8-16	R^2 = 0.02, F (1,53) = 1.225, p=0.273, q=0.425
Tempo test	16.13 (5.79)	6-30	18.04 (3.44)	12-26	R^2 = 0.04, F (1,53) = 2.01, p=0.162, q=0.324
Flanker processing speed	989.96 (182.86)	725-1273.56	902.8 (181.27)	581.94-1197.63	R^2 = 0.05, F (1,53) = 3.062, p=0.086, q=0.2
Flanker inhibition	56.7 (82.44)	-111.94-203.69	382.7 (1437.5)	-108.81-5246.50	R^2 = 0.04, F (1,54) = 1.649, p=0.205, q=0.358
Reality filtering, TCC	0.02 (0.06)	-0.11-0.2	0.01 (0.08)	-0.11-0.21	R^2 = 0.003, F (1,53) = 0.151, p=0.699, q=0.752
SDQ total	12.03 (5.13)	2-24	7.32 (5.69)	1-22	R^2 = 0.16, F (1,52) = 10.081, p=0.003, q=0.009
KIDSCREEN total	107.94 (11.74)	85-127	104.24 (15.1)	60-124	R^2 = 0.02, F (1,50) = 0.985, p=0.326, q=0.456
Social goal	3.91 (0.55)	2.43-4.71	3.87 (0.47)	2.86-4.5	R^2 = 0.002, F (1,51) = 0.099, p=0.754, q=0.754
Self-compassion	2.86 (0.52)	1.75-3.75	3.2 (0.52)	2.67-4.42	R^2 = 0.10, F (1,50) = 5.464, p=0.023, q=0.066
Affect recognition	10.22 (2.85)	4-15	10.52 (2.02)	5-15	R^2 = 0.004, F (1,53) = 0.191, p=0.664, q=0.752
Theory of mind	24.56 (1.83)	21-27	24.78 (1.59)	22-27	R^2 = 0.004, F (1,53) = 0.215, p=0.645, q=0.752

Note: Differences between the VPT and FT groups were examined using linear model. All p-values that survived false discovery rate (FDR) correction (threshold: q < 0.1) are indicated in bold.

Supplementary Table S4. Group comparison on neurobehavioural outcome measures of the VPT and FT young adolescents, including the General Ability Index (GAI) as covariate. Linear regression models included each neurobehavioural measures as outcome variable and the group as a fixed effect (i.e., VPT and FT) and GAI as covariate.

	Linear regression model	Explanatory variable: group	Explanatory variable: GAI
BRIEF CEG	R^2 =0.326, F (3,50) = 12.092, p = 0, q = 0.001	t (51) = -4.455, p < 0.001	t (51) = -0.597, p = 0.553
BRIEF MI	R^2 =0.296, F (3,50) = 10.504, p = 0, q = 0.001	t (51) = -4.135, p < 0.001	t (51) = -0.597, p = 0.553
BRIEF BRI	R^2 =0.181, F (3,50) = 5.515, p = 0.007, q = 0.019	t (51) = -2.802, p = 0.007	t (51) = -0.824, p = 0.414
Letter-Number sequencing	R^2 =0.194, F (3,52) = 6.239, p = 0.004, q = 0.013	t (53) = -0.017, p = 0.986	t (53) = 3.32, p = 0.002
Tempo test	R^2 =0.055, F (3,52) = 1.511, p = 0.23, q = 0.322	t (53) = 0.982, p = 0.331	t (53) = 1.005, p = 0.319
Flanker processing speed	R^2 =0.086, F (3,52) = 2.439, p = 0.097, q = 0.194	t (53) = -1.101, p = 0.276	t (53) = -1.331, p = 0.189
Flanker inhibition	R^2 =0.041, F (3,52) = 1.118, p = 0.335, q = 0.426	t (53) = 1.344, p = 0.185	t (53) = 0.078, p = 0.938
Reality filtering, TCC	R^2 =0.004, F (3,52) = 0.108, p = 0.897, q = 0.897	t (53) = -0.253, p = 0.801	t (53) = -0.261, p = 0.795
SDQ total	R^2 =0.216, F (3,51) = 7.04, p = 0.002, q = 0.009	t (52) = -2.466, p = 0.017	t (52) = -1.874, p = 0.067
KIDSCREEN total	R^2 =0.07, F (3,49) = 1.834, p = 0.171, q = 0.265	t (50) = -1.425, p = 0.161	t (50) = 1.628, p = 0.11
Social goal	R^2 =0.074, F (3,50) = 1.985, p = 0.148, q = 0.259	t (51) = -0.935, p = 0.354	t (51) = 1.966, p = 0.055
Self-compassion	R^2 =0.146, F (3,49) = 4.181, p = 0.021, q = 0.049	t (50) = 1.801, p = 0.078	t (50) = 1.647, p = 0.106
Affect recognition	R^2 =0.011, F (3,52) = 0.278, p = 0.758, q = 0.816	t (53) = 0.197, p = 0.844	t (53) = 0.607, p = 0.547
Theory of mind	R^2 =0.015, F (3,52) = 0.39, p = 0.679, q = 0.792	t (53) = 0.173, p = 0.864	t (53) = 0.752, p = 0.455

Supplementary Table S5. Comparison of neurobehavioural scores before and after MBI intervention in VPT young adolescents. Linear regression models included each neurobehavioural measures that showed a significant difference before and after MBI as outcome variable, before / after MBI as a fixed effect, and subject as a random effect.

	Before MBI		After MBI		Group comparisons
	Mean (SD)	Range	Mean (SD)	Range	
BRIEF CEG	63.1 (9.66)	40-79	56.5 (9.95)	37-73	R^2 = 0.71, F (1,29.27) = 21.741, p<0.001, q<0.001
BRIEF MI	61.94 (8.45)	42-78	56.87 (11.65)	37-86	R^2 = 0.50, F (1,29.9) = 7.278, p=0.011, q=0.019
BRIEF BRI	61.61 (14.21)	38-98	56.5 (12.29)	38-90	R^2 = 0.64, F (1,29.76) = 6.034, p=0.039, q=0.025
SDQ total	12.03 (5.13)	2-24	9.57 (4.15)	4-18	R^2 = 0.63, F (1,29.95) = 10.748, p=0.003, q=0.007
Self-compassion	2.86 (0.52)	1.75-3.75	3.07 (0.58)	1.83-4.1	R^2 = 0.52, F (1,28.61) = 4.009, p=0.055, q=0.055

Note: R^2 correspond to conditional R^2; All p-values that survived false discovery rate (FDR) correction (threshold: q < 0.1) are indicated in bold.

Supplementary Table S6. Group comparison on neurobehavioural outcome measures of the VPT after MBI and FT young adolescents. Linear regression models included each neurobehavioural measures as outcome variable and the group as a fixed effect (i.e., VPT after MBI and FT).

	VPT after MBI		FT		Group comparisons
	Mean (SD)	Range	Mean (SD)	Range	
BRIEF CEG	56.5 (9.95)	37-73	48.86 (11.36)	36-73	R^2 = 0.117, F (1,50) = 6.627, p =0.013, q = 0.183
BRIEF MI	56.87 (11.65)	37-86	50 (10.53)	36-69	R^2 = 0.087, F (1,50) = 4.776, p =0.034, q = 0.2
BRIEF BRI	56.5 (12.29)	38-90	48.86 (14.12)	36-81	R^2 = 0.08, F (1,50) = 4.322, p =0.043, q = 0.2
Letter-Number sequencing	11.28 (2.85)	6-17	11.26 (2.34)	8-16	R^2 = 0.0007, F (1,53) = 0.001, p =0.978, q = 0.988
Tempo test	18.06 (5.76)	8-33	18.04 (3.44)	12-26	R^2 = 0.0002, F (1,52) = 0, p =0.988, q = 0.988
Flanker processing speed	939.39 (160.04)	610.94-1298	902.8 (181.27)	581.94-1197.63	R2 = 0.012, F (1,53) = 0.626, p =0.432, q = 0.55
Flanker inhibition	31.94 (74.2)	-94.75-214.88	832.05 (2918.73)	-108.81-5246.50	R2 = 0.044, F (1,53) = 2.42, p =0.126, q = 0.293
Reality filtering, TCC	0.06 (0.15)	-0.26-0.65	0.01 (0.08)	-0.11-0.21	R^2 = 0.038, F (1,53) = 2.077, p =0.155, q = 0.311
SDQ total	9.57 (4.15)	4-18	7.32 (5.69)	1-22	R^2 = 0.052, F (1,50) = 2.723, p =0.105, q = 0.293
KIDSCREEN total	108.37 (8.58)	88-125	104.24 (15.1)	60-124	R^2 = 0.031, F (1,49) = 1.542, p =0.22, q = 0.343
Social goal	3.96 (0.53)	2.93-4.64	3.87 (0.47)	2.86-4.5	R^2 = 0.008, F (1,50) = 0.428, p =0.516, q = 0.602
Self-compassion	3.07 (0.58)	1.83-4.1	3.2 (0.52)	2.67-4.42	R^2 = 0.014, F (1,49) = 0.696, p =0.408, q = 0.55
Affect recognition	11.28 (2.22)	5-16	10.52 (2.02)	5-15	R^2 = 0.031, F (1,53) = 1.689, p =0.199, q = 0.343
Theory of mind	25.53 (1.67)	22-28	24.78 (1.59)	22-27	R^2 = 0.05, F (1,53) = 2.802, p =0.1, q = 0.293

Note: Differences between the VPT-after MBI and FT groups were examined using linear model. All p-values that survived false discovery rate (FDR) correction (threshold: q < 0.1) are indicated in bold.

Supplementary Table S7. iCAPs functional networks of regions from the automated anatomical labelling 2 (AAL2) atlas. Percentiles indicate the fraction of voxels of a functional network or region that have a z-score > 2. A network/region is listed if more than 20% of the network/region is included in the iCAP.

iCAP	Lobe	Region	Percentile	Mean z-score	Voxels
**iCAP 1**	Posterior Fossa	Cerebelum_10_R	100	2	6
	Frontal	Olfactory_L	96.93	3.18	221
	Frontal	Olfactory_R	94.26	2.78	230
	Subcortical grey nucleus	Caudate_L	91.97	2.89	584
	Frontal	OFCmed_L	87.96	2.75	336
	Posterior	Vermis_9	86.81	2.65	125
	Frontal	Rectus_L	86.17	2.84	461
	Subcortical grey nucleus	Caudate_R	85.38	2.52	578
	Frontal	Rectus_R	84.92	2.74	428
	Posterior Fossa	Cerebelum_9_L	81.44	2.4	79
	Posterior Fossa	Cerebelum_9_R	81.32	2.66	74
	Subcortical grey nucleus	Thalamus_L	79.26	2.34	428
	Frontal	OFCmed_R	78.71	2.41	366
	Subcortical grey nucleus	Thalamus_R	74.95	2.23	401
	Subcortical grey nucleus	Putamen_L	69.4	2.31	517
	Posterior Fossa	Vermis_10	60	2.39	6
	Posterior Fossa	Vermis_1_2	58.33	2.11	14
	Frontal	OFCpost_L	53.06	2.15	243
	Subcortical grey nucleus	Pallidum_L	52.05	2.22	38
	Temporal	Temporal_Pole_Mid_L	51.43	2.18	90
	Limbic	Cingulate_Ant_L	48.78	2.16	540
	Limbic	Cingulate_Mid_L	40.11	1.97	586
	Temporal	Temporal_Inf_L	37.38	2.18	680
	Limbic	Cingulate_Ant_R	35.79	2.06	350
	Frontal	Frontal_Med_Orb_L	35.59	2.2	158
	Frontal	Frontal_Med_Orb_R	32.52	2.24	174
	Temporal	Temporal_Inf_R	27.3	2.26	507
	Temporal	Temporal_Pole_Sup_L	21.08	2.07	141
**iCAP2**	Frontal	Frontal_Inf_Tri_R	83.14	2.42	779

	Frontal	Frontal_Inf_Tri_L	83.05	2.29	1029
	Frontal	Frontal_Sup_Medial_R	71.82	2.21	836
	Frontal	OFClat_R	71.58	2.16	68
	Central	Frontal_Inf_Orb_2_R	66.74	2.16	285
	Frontal	OFClat_L	64.94	2.08	100
	Frontal	Frontal_Sup_Medial_L	63.58	2.26	864
	Frontal	Frontal_Sup_2_L	54.2	2.44	1251
	Frontal	Frontal_Mid_2_R	52.07	2.21	1308
	Frontal	Frontal_Mid_2_L	51.54	2.38	1157
	Frontal	Frontal_Sup_2_R	46.12	2.37	1110
	Temporal	Temporal_Pole_Sup_R	37.69	2.01	271
	Frontal	OFCpost_R	20.6	2.09	76
**iCAP3**	Subcortical grey nucleus	Amygdala_R	89.58	2.91	172
	Subcortical grey nucleus	Amygdala_L	85.94	2.25	165
	Posterior Fossa	Vermis_3	78.9	2.33	86
	Temporal	Temporal_Pole_Sup_R	75.52	2.75	543
	Limbic	ParaHippocampal_R	66.58	2.5	492
	Posterior Fossa	Vermis_1_2	62.5	2.6	15
	Limbic	ParaHippocampal_L	58.18	2.53	384
	Temporal	Temporal_Pole_Mid_R	55.19	2.01	170
	Limbic	Hippocampus_R	51.66	2.15	311
	Temporal	Temporal_Pole_Sup_L	42.45	2.37	284
	Limbic	Hippocampus_L	41.01	2.23	276
	Limbic	Insula_R	37.62	2.32	544
	Posterior Fossa	Vermis_8	32.14	2.02	63
	Frontal	Olfactory_R	21.72	2.34	53
	Temporal	Temporal_Sup_L	21.4	2.03	309
	Temporal	Temporal_Sup_R	19.95	2.18	374
	Occipital	Fusiform_R	19.43	2.47	333
	Occipital	Fusiform_L	19.29	2.74	308

	Limbic	Insula_L	18.29	2.08	282
	Frontal	Olfactory_L	16.67	2.02	38
**iCAP4**	Occipital	Occipital_Sup_R	80.43	2.62	378
	Occipital	Cuneus_L	79.54	2.31	692
	Occipital	Occipital_Sup_L	70.82	2.33	301
	Parietal	Angular_R	70.8	2.43	679
	Occipital	Occipital_Mid_R	67.16	2.69	634
	Occipital	Cuneus_R	66.94	2.17	401
	Parietal	Angular_L	64.26	2.16	489
	Occipital	Occipital_Mid_L	64.09	2.65	864
	Parietal	SupraMarginal_R	49.67	2.04	521
	Parietal	Precuneus_R	29.39	2.11	524
	Occipital	Calcarine_R	28.43	1.73	257
	Parietal	Parietal_Sup_R	25.46	2.11	207
	Parietal	Parietal_Sup_L	23.3	2.12	195
	Parietal	Precuneus_L	23.05	2.19	423
	Temporal	Temporal_Mid_R	17.05	2.28	445
**iCAP 5**	Posterior Fossa	Cerebelum_7b_R	98.08	2.18	51
	Posterior Fossa	Vermis_8	96.94	2.69	190
	Posterior Fossa	Cerebelum_6_L	95.96	2.48	1545
	Posterior Fossa	Cerebelum_6_R	92.1	2.65	1527
	Posterior Fossa	Vermis_9	91.67	2.43	132
	Posterior Fossa	Vermis_7	90.96	2.54	151
	Posterior Fossa	Cerebelum_8_L	89.47	2.55	102
	Posterior Fossa	Cerebelum_Crus1_L	87.16	2.59	1752
	Posterior Fossa	Vermis_6	85.99	2.65	270
	Posterior Fossa	Cerebelum_9_L	85.57	2.26	83
	Posterior Fossa	Cerebelum_Crus1_R	85.32	2.59	1616
	Posterior Fossa	Cerebelum_7b_L	83.53	2.11	71
	Posterior Fossa	Cerebelum_8_R	82.98	2.21	117

	Posterior Fossa	Cerebelum_Crus2_R	80.19	2.3	753
	Posterior Fossa	Cerebelum_Crus2_L	77.42	2.3	902
	Posterior Fossa	Cerebelum_9_R	76.92	2.27	70
	Occipital	Fusiform_R	43.41	2.27	744
	Occipital	Fusiform_L	42.39	2.15	677
	Occipital	Occipital_Inf_R	40.44	2.09	129
	Posterior Fossa	Vermis_1_2	37.5	2.04	9
	Occipital	Lingual_R	32.75	2.11	428
**iCAP6**	Frontal	Paracentral_Lobule_R	91.81	2.38	258
	Frontal	Supp_Motor_Area_L	91.58	2.92	870
	Frontal	Supp_Motor_Area_R	90.67	2.83	952
	Frontal	Paracentral_Lobule_L	90.61	3.08	434
	Central	Precentral_L	47.4	2.95	746
	Central	Precentral_R	38.38	2.67	608
	Central	Postcentral_R	35.91	2.46	572
	Central	Postcentral_L	35.8	2.29	668
	Parietal	Parietal_Sup_L	34.65	2.04	290
	Frontal	Frontal_Sup_2_R	32.78	2.72	789
	Frontal	Frontal_Sup_2_L	24.83	2.69	573
	Parietal	Parietal_Sup_R	20.91	2.01	170
	Parietal	Precuneus_L	14.6	2.15	268
**iCAP7**	Occipital	Occipital_Inf_R	82.13	3.09	262
	Posterior Fossa	Cerebelum_Crus2_R	74.12	3.73	696
	Occipital	Occipital_Inf_L	70.25	2.9	255
	Posterior Fossa	Cerebelum_Crus2_L	69.87	3.98	814
	Posterior Fossa	Vermis_7	57.83	2.14	96
	Posterior Fossa	Cerebelum_7b_R	57.69	2.06	30
	Posterior Fossa	Vermis_8	48.47	2.48	95
	Posterior Fossa	Cerebelum_Crus1_R	45.78	3.12	867
	Posterior Fossa	Cerebelum_Crus1_L	42.54	3.31	855

	Posterior Fossa	Cerebelum_7b_L	40	2.12	34
	Temporal	Temporal_Inf_R	30.86	2.13	573
	Occipital	Occipital_Mid_L	24.11	2.57	325
	Occipital	Calcarine_L	22.21	2.75	315
	Posterior Fossa	Cerebelum_8_L	16.67	2.05	19
	Posterior Fossa	Vermis_6	16.56	1.78	52
	Occipital	Lingual_R	16.07	3.65	210
	Occipital	Occipital_Mid_R	13.35	2.15	126
	Occipital	Fusiform_L	12.9	2.25	206
	Occipital	Lingual_L	11.98	3.56	148
	Occipital	Occipital_Sup_L	10.35	2.7	44
**iCAP8**	Frontal	OFCant_L	99.67	4.88	302
	Frontal	OFClat_L	99.35	4.96	153
	Frontal	OFClat_R	98.95	4.89	94
	Frontal	OFCant_R	98.7	5.5	381
	Frontal	OFCmed_R	80	3.18	372
	Frontal	OFCmed_L	75.13	2.88	287
	Frontal	OFCpost_R	74.8	3.03	276
	Frontal	OFCpost_L	71.4	2.73	327
	Central	Frontal_Inf_Orb_2_R	59.48	2.46	254
	Frontal	Frontal_Med_Orb_R	44.3	2.62	237
	Frontal	Frontal_Inf_Orb_2_L	43.51	2.3	201
	Frontal	Rectus_R	39.68	2.66	200
	Frontal	Frontal_Med_Orb_L	38.74	2.75	172
	Frontal	Rectus_L	38.69	2.17	207
	Frontal	Frontal_Sup_2_L	29.03	2.99	670
	Frontal	Frontal_Sup_2_R	29	3.01	698
	Frontal	Frontal_Sup_Medial_R	21.65	2.15	252
	Frontal	Frontal_Mid_2_R	20.62	3.55	518
	Frontal	Frontal_Mid_2_L	20.49	3.3	460

Supplementary Table S8. Bootstrapping mean and standard deviations of the loadings for clinical and reliable change in the total duration of each iCAPs of the PLSC analyses.

**LC1 – Loadings for clinical measures and RCI of the total duration for each iCAPs**			
	**Clinical loadings**		
		**Measures**	**Loadings, bootstrapping mean (bootstrapping standard deviation)**
		Gestational age	-0.348 (0.193)
		Age at testing	-0.241 (0.229)
		RCI BRIEF CEG	-0.924 (0.044)
		RCI BRIEF MI	-0.716 (0.093)
		RCI BRIEF BRI	-0.783 (0.092)
		RCI SDQ total	-0.463 (0.186)
		RCI Self-compassion	0.054 (0.285)
		Gestational age	-0.348 (0.193)
	**RCI of the total duration of each iCAPs loadings**		
		**Measures**	**Loadings, bootstrapping mean (bootstrapping standard deviation)**
		RCI total duration iCAP1	0.728 (0.131)
		RCI total duration iCAP2	0.834 (0.072)
		RCI total duration iCAP3	0.903 (0.04)
		RCI total duration iCAP4	0.817 (0.088)
		RCI total duration iCAP5	0.083 (0.211)
		RCI total duration iCAP6	0.492 (0.181)
		RCI total duration iCAP7	-0.355 (0.126)
		RCI total duration iCAP8	0.274 (0.153)

Note: RCI, reliable change index

Supplementary Table S9. Comparison of before and after MBI in VPT participants for static inter-iCAPs correlations. Linear regression models showed no significant difference in static functional connectivity before and after MBI.

**Inter-iCAPs**	**Comparision before MBI and after MBI**
iCAP2 & iCAP1	R^2 = 0.002, F (1,62) = 0.094, p = 0.76, q = 0.871
iCAP3 & iCAP1	R^2 = 0.006, F (1,62) = 0.361, p = 0.55, q = 0.715
iCAP3 & iCAP2	R^2 = 0.018, F (1,62) = 1.143, p = 0.289, q = 0.715
iCAP4 & iCAP1	R^2 = 0.0002, F (1,62) = 0.012, p = 0.912, q = 0.912
iCAP4 & iCAP2	R^2 = 0.005, F (1,62) = 0.34, p = 0.562, q = 0.715
iCAP4 & iCAP3	R^2 = 0.001, F (1,62) = 0.059, p = 0.808, q = 0.871
iCAP5 & iCAP1	R^2 = 0.053, F (1,62) = 3.487, p = 0.067, q = 0.715
iCAP5 & iCAP2	R^2 = 0.006, F (1,62) = 0.354, p = 0.554, q = 0.715
iCAP5 & iCAP3	R^2 = 0.007, F (1,62) = 0.447, p = 0.506, q = 0.715
iCAP5 & iCAP4	R^2 = 0.014, F (1,62) = 0.852, p = 0.36, q = 0.715
iCAP6 & iCAP1	R^2 = 0.022, F (1,62) = 1.426, p = 0.237, q = 0.715
iCAP6 & iCAP2	R^2 = 0.01, F (1,62) = 0.643, p = 0.426, q = 0.715
iCAP6 & iCAP3	R^2 = 0.009, F (1,62) = 0.59, p = 0.445, q = 0.715
iCAP6 & iCAP4	R^2 = 0.015, F (1,62) = 0.947, p = 0.334, q = 0.715
iCAP6 & iCAP5	R^2 = 0.002, F (1,62) = 0.094, p = 0.76, q = 0.871
iCAP7 & iCAP1	R^2 = 0.006, F (1,62) = 0.361, p = 0.55, q = 0.715
iCAP7 & iCAP2	R^2 = 0.018, F (1,62) = 1.143, p = 0.289, q = 0.715
iCAP7 & iCAP3	R^2 = 0.0002, F (1,62) = 0.012, p = 0.912, q = 0.912
iCAP7 & iCAP4	R^2 = 0.005, F (1,62) = 0.34, p = 0.562, q = 0.715
iCAP7 & iCAP5	R^2 = 0.001, F (1,62) = 0.059, p = 0.808, q = 0.871
iCAP7 & iCAP6	R^2 = 0.053, F (1,62) = 3.487, p = 0.067, q = 0.715
iCAP8 & iCAP1	R^2 = 0.006, F (1,62) = 0.354, p = 0.554, q = 0.715
iCAP8 & iCAP2	R^2 = 0.007, F (1,62) = 0.447, p = 0.506, q = 0.715
iCAP8 & iCAP3	R^2 = 0.014, F (1,62) = 0.852, p = 0.36, q = 0.715
iCAP8 & iCAP4	R^2 = 0.022, F (1,62) = 1.426, p = 0.237, q = 0.715
iCAP8 & iCAP5	R^2 = 0.01, F (1,62) = 0.643, p = 0.426, q = 0.715
iCAP8 & iCAP6	R^2 = 0.009, F (1,62) = 0.59, p = 0.445, q = 0.715
iCAP8 & iCAP7	R^2 = 0.015, F (1,62) = 0.947, p = 0.334, q = 0.715

Supplementary Figures

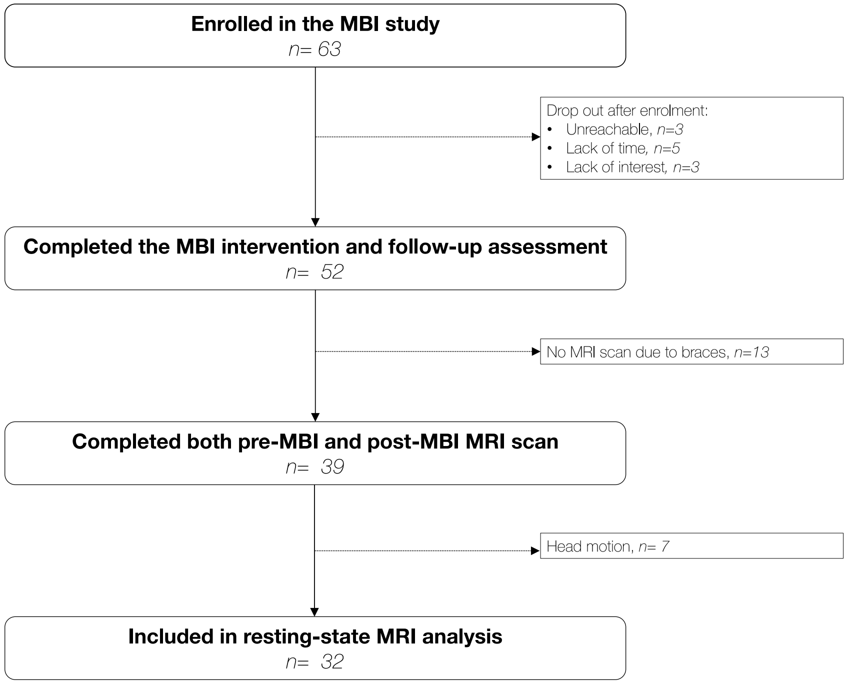

Supplementary Figure S1. Participant flow chart. All neuropsychological assessments and MRI acquisitions were completed at the Campus Biotech in Geneva, Switzerland. MBI, Mindfulness-based intervention; MRI, magnetic resonance imaging.

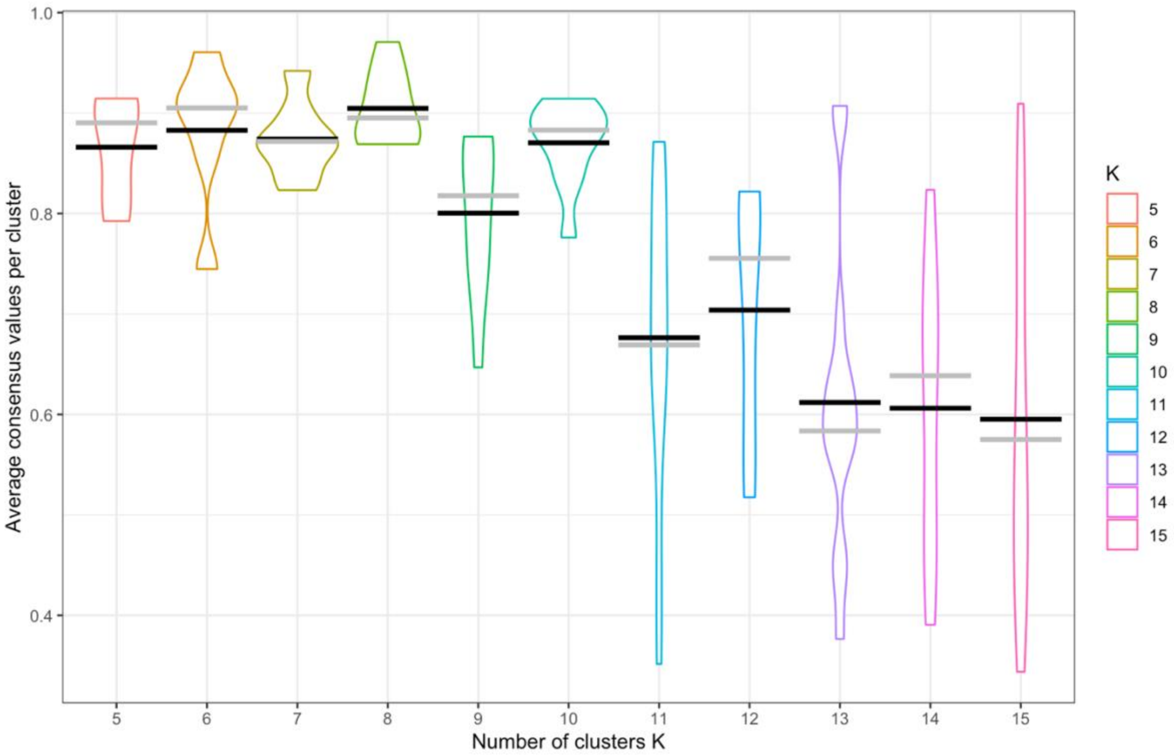

Supplementary Figure S2. Consensus clustering quality measures for cluster numbers K = 5 to K = 15. The distribution of average consensus values per cluster: the mean for each cluster is represented by a black line and the median is represented by a grey line. Mean consensus is maximal (closest to 1) for K = 8.

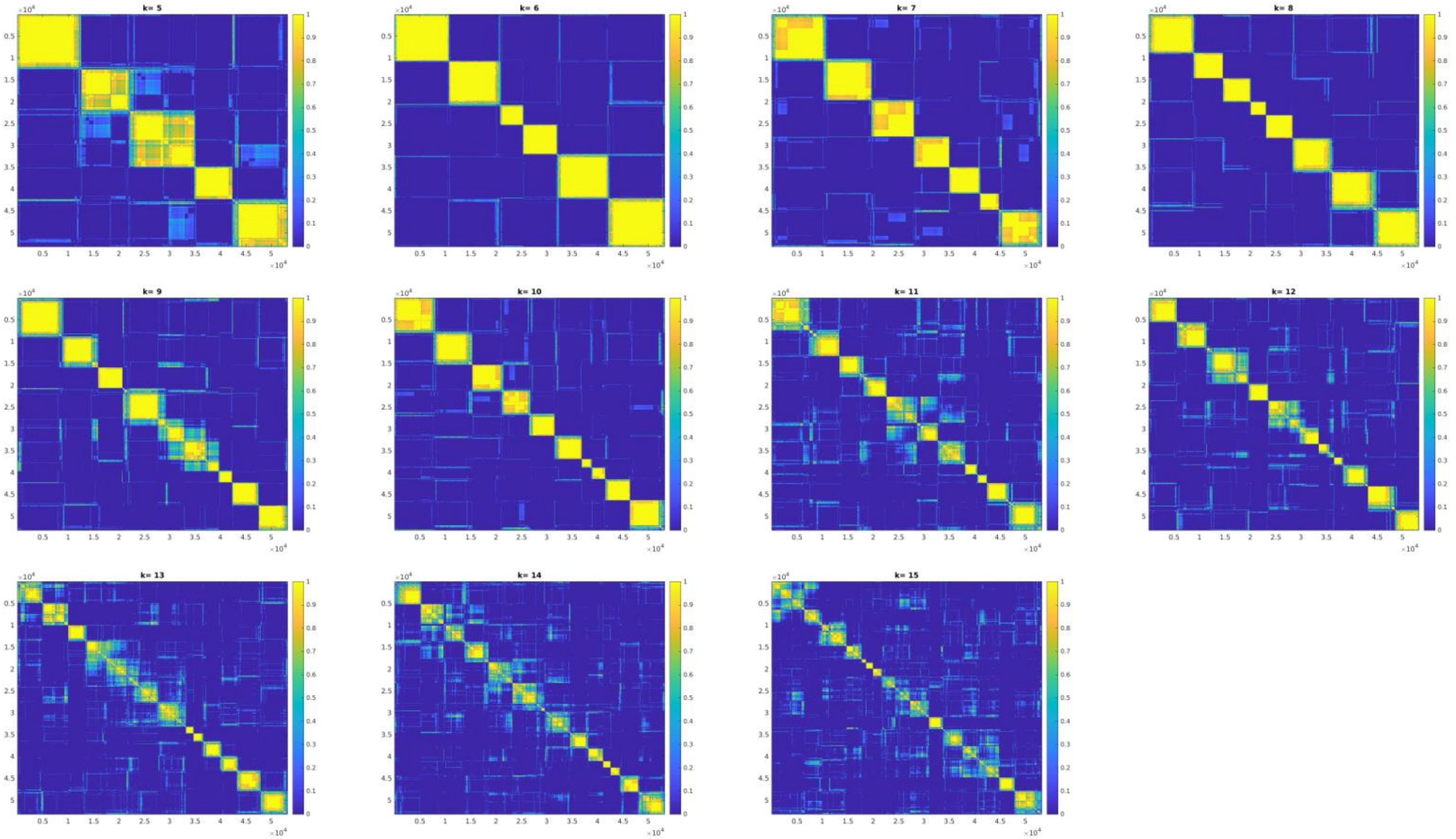

Supplementary Figure S3. Consensus matrices for cluster numbers K = 5 to K = 15. High values in the matrix indicate that the two corresponding frames were clustered together during re-sampling. A value of 1 means that the frames were always clustered together, while a value of 0 means that the frames were never clustered together. We selected to use K = 8 based on visual inspection of the consensus matrices and evaluation of consensus clustering quality measures (Monti et al., 2003). In Supplementary Figure S1, we plot the distribution of average consensus values per cluster to assess the quality of the clustering for each K.

REFERENCES

1. Kabat-Zinn J. Mindfulness-Based interventions in context: Past, present and future. Clinical Psychology: Science and Practice 2003; 10: 144-156.
2. Segal ZV, Williams JMG, Teasdale JD. Mindfulness-based cognitive theraphy for depression: a new approach to preventing relapse. Guilford Publications, 2001.
3. Tamnes CK, Roalf DR, Goddings A-L, Lebel C. Diffusion MRI of white matter microstructure development in childhood and adolescence: Methods, challenges and progress. Developmental cognitive neuroscience 2018; 33: 161-175.
4. de Bie HM, Boersma M, Wattjes MP et al. Preparing children with a mock scanner training protocol results in high quality structural and functional MRI scans. 2010; 169: 1079-1085.
5. Freitas LGA, Liverani MC, Siffredi V et al. Altered orbitofrontal activation in preterm-born young adolescents during performance of a reality filtering task. NeuroImage: Clinical 2021; 30: 102668.
6. Liverani MC, Freitas LGA, Siffredi V et al. Get real: Orbitofrontal cortex mediates the ability to sense reality in early adolescents. Brain and Behavior 2020.
7. Hutton C, Bork A, Josephs O, Deichmann R, Ashburner J, Turner R. Image distortion correction in fMRI: a quantitative evaluation. NeuroImage 2002; 16: 217-240.
8. Ashburner J, Friston KJ. Unified segmentation. NeuroImage 2005; 26: 839-851.
9. Ashburner J. A fast diffeomorphic image registration algorithm. Neuroimage 2007; 38: 95-113.
10. Power JD, Barnes KA, Snyder AZ, Schlaggar BL, Petersen SE. Spurious but systematic correlations in functional connectivity MRI networks arise from subject motion. NeuroImage 2012; 59: 2142-54.
11. Power JD, Mitra A, Laumann TO, Snyder AZ, Schlaggar BL, Petersen SE. Methods to detect, characterize, and remove motion artifact in resting state fMRI. NeuroImage 2014; 84: 320-41.
12. Karahanoğlu FI, Van De Ville D. Transient brain activity disentangles fMRI resting-state dynamics in terms of spatially and temporally overlapping networks. Nature communications 2015; 6: 1-10.
13. Zöller D, Sandini C, Karahanoğlu FI et al. Large-scale brain network dynamics provide a measure of psychosis and anxiety in 22q11. 2 deletion syndrome. Biological Psychiatry: Cognitive Neuroscience
Neuroimaging 2019; 4: 881-892.
14. Farouj Y, Karahanoğlu FI, Van De Ville D. Regularized spatiotemporal deconvolution of fMRI data using gray-matter constrained total variation. 2017 IEEE 14th International Symposium On Biomedical Imaging (Isbi 2017). Ieee, 2017; 472-475.
15. Karahanoğlu FI, Caballero-Gaudes C, Lazeyras F, Van De Ville D. Total activation: fMRI deconvolution through spatio-temporal regularization. NeuroImage 2013; 73: 121-134.
16. Monti S, Tamayo P, Mesirov J, Golub T. Consensus clustering: a resampling-based method for class discovery and visualization of gene expression microarray data. Machine learning 2003; 52: 91-118.
17. Zöller DM, Bolton TA, Karahanoğlu FI, Eliez S, Schaer M, Van De Ville D. Robust recovery of temporal overlap between network activity using transient-informed spatio-temporal regression. IEEE Transactions on Medical Imaging 2018; 38: 291-302.
18. Bommarito G, Tarun A, Farouj Y et al. Altered anterior default mode network dynamics in progressive multiple sclerosis. Multiple Sclerosis Journal 2021: 13524585211018116.
19. Piguet C, Karahanoğlu FI, Saccaro LF, Van De Ville D, Vuilleumier P. Mood disorders disrupt the functional dynamics, not spatial organization of brain resting state networks. NeuroImage: Clinical 2021; 32: 102833.

20. Siffredi V, Farouj Y, Tarun A et al. Large-scale functional network dynamics in human callosal agenesis: Increased subcortical involvement and preserved laterality. NeuroImage 2021; 243: 118471.
21. Jacobson N, Truax P. Clinical significance: A statistical approach to defining meaningful change in psychotherapy research. Journal of consulting and clinical psychology 1991; 59: 12–19.
22. McIntosh AR, Lobaugh NJJN. Partial least squares analysis of neuroimaging data: applications and advances. 2004; 23: S250-S263.
23. Kebets V, Holmes AJ, Orban C et al. Somatosensory-Motor Dysconnectivity Spans Multiple Transdiagnostic Dimensions of Psychopathology. Biol Psychiat 2019; 0.
24. Zoller D, Sandini C, Karahanoglu FI et al. Large-Scale Brain Network Dynamics Provide a Measure of Psychosis and Anxiety in 22q11.2 Deletion Syndrome. Biological Psychiatry: Cognitive Neuroscience and Neuroimaging 2019; 4: 881-892
25. Sherry A, Henson RK. Conducting and interpreting canonical correlation analysis in personality research: A user-friendly primer. Journal of personality assessment 2005; 84: 37-48.
26. Garrett DD, Kovacevic N, McIntosh AR, Grady CL. Blood oxygen level-dependent signal variability is more than just noise. Journal of Neuroscience 2010; 30: 4914-4921.
27. Gioia G, Isquith P, Guy S, Kenworthy L. BRIEF – Behavior Rating Inventory of Executive Function. Professional manual. Psychological Assessment Resources Inc, Odessa, FL, 2000.
28. Wechsler D. Manual for the Wechsler Intelligence Scale for Children-IV. Psychological Corporation, New York, 2003.
29. De Vos T. TTR: Tempotest rekenen. Swets & Zeitlinger, Lisse, The Netherlands 1992.
30. Christ SE, Kester LE, Bodner KE, Miles JH. Evidence for selective inhibitory impairment in individuals with autism spectrum disorder. Neuropsychology 2011; 25: 690-701.
31. Liverani MC, Manuel AL, Nahum L, Guardabassi V, Tomasetto C, Schnider A. Children's sense of reality: The development of orbitofrontal reality filtering. Child neuropsychology : a journal on normal and abnormal development in childhood and adolescence 2017; 23: 408-421.
32. Schnider A. The confabulating mind : how the brain creates reality. Oxford University Press, Oxford ; New York, 2018.
33. Goodman R. Psychometric properties of the strengths and difficulties questionnaire. J Am Acad Child Adolesc Psychiatry 2001; 40: 1337-45.
34. Robitail S, Ravens-Sieberer U, Simeoni MC et al. Testing the structural and cross-cultural validity of the KIDSCREEN-27 quality of life questionnaire. Qual Life Res 2007; 16: 1335-45.
35. Patrick H, Hicks L, Ryan AM. Relations of perceived social efficacy and social goal pursuit to self-efficacy for academic work. Journal of Early Adolescence 1997: 109-128.
36. Raes F, Pommier E, Neff KD, Van Gucht D. Construction and factorial validation of a short form of the Self-Compassion Scale. Clin Psychol Psychother 2011; 18: 250-5.
37. Korkman M, Kirk U, Kemp S. NEPSY-II: A developmental neuropsychological assessment, second edition. San Antonio, TX, 2007.
